# A Randomized Clinical Trial of the Immunogenicity of 7-Valent Pneumococcal Conjugate Vaccine Compared to 23-Valent Polysaccharide Vaccine in Frail, Hospitalized Elderly

**DOI:** 10.1371/journal.pone.0094578

**Published:** 2014-04-23

**Authors:** C. Raina MacIntyre, Iman Ridda, Zhanhai Gao, Aye M. Moa, Peter B. McIntyre, John S. Sullivan, Thomas R. Jones, Andrew Hayen, Richard I. Lindley

**Affiliations:** 1 School of Public Health and Community Medicine, UNSW Australia, The University of New South Wales, Sydney, Australia; 2 National Centre for Immunization Research and Surveillance (NCIRS), Westmead, Australia; 3 Central Clinical School, The University of Sydney, Sydney, Australia; 4 Pfizer Vaccine Research, Pfizer, Pearl River, New York, United States of America; 5 Westmead Clinical School, Westmead Hospital, and the George Institute for Global Health, The University of Sydney, Sydney, Australia; Public Health England, United Kingdom

## Abstract

**Background:**

Elderly people do not mount strong immune responses to vaccines. We compared 23-valent capsular polysaccharide (23vPPV) alone versus 7-valent conjugate (PCV7) vaccine followed by 23vPPV 6 months later in hospitalized elderly.

**Methods:**

Participants were randomized to receive 23vPPV or PCV7-23vPPV. Antibodies against serotypes 3, 4, 6A, 6B, 9V, 14, 18C, 19A, 19F, 23F were measured by enzyme-linked immunosorbent (ELISA) and opsonophagocytic (OPA) assays at baseline, 6 months and 12 months.

**Results:**

Of 312 recruited, between 40% and 72% of subjects had undetectable OPA titres at baseline. After one dose, PCV7 recipients had significantly higher responses to serotypes 9V (both assays) and 23F (OPA only), and 23vPPV recipients had significantly higher responses to serotype 3 (ELISA), 19F and 19A (OPA only). In subjects with undetectable OPA titres at baseline, a proportionately greater rise in OPA titre (P<0.01) was seen for all serotypes after both vaccines. The GMT ratio of OPA was significantly higher at 12 months in the PCV7-23vPPV group for serotypes 6A, 9V, 18C and 23F. OPA titre levels for these serotypes increased moderately after 6 months, whereas immunity waned in the 23vPPV only arm.

**Conclusion:**

We did not show overwhelming benefit of one vaccine over the other. Low baseline immunity does not preclude a robust immune response, reiterating the importance of vaccinating the frail elderly. A schedule of PCV7-23vPPV prevents waning of antibody, suggesting that both vaccines could be useful in the elderly. Follow up studies are needed to determine persistence of immunity.

**Trial Registration:**

The Australian Clinical Trials Registry ACTRN12607000387426

## Background

*Streptococcus pneumoniae* causes invasive pneumococcal disease (IPD), with peak incidence in the very young and the very old [1]. In contrast to children, over 80% of adults with IPD have underlying risk factors [1], [2]. Although 7-valent and more recently 13-valent pneumococcal conjugate vaccines (PCV7 and PCV13), are used in infant immunization programs in many countries, only 23-valent capsular polysaccharide vaccine (23vPPV) is recommended for adults.

In Australia, a funded national immunization program for both 23vPPV in adults ≥65 years and PCV7 in children under 2 years was introduced in 2005. Infant programs have resulted in significant reductions in IPD due to vaccine serotypes in all age groups, including adults over 65 years [3]–[5]. There has been little reduction in IPD serotypes specific to 23vPPV in the population aged >65 years. 23vPPV is 60–70% effective against IPD, with declining effectiveness and waning immunity in older adults [6], [7].

A number of studies (*Table 1*) have compared the immunogenicity of PCV7 and 23vPPV in adults [8]–[16]. These studies differ in study populations, age of subjects, vaccine schedule and intervals of follow up, making comparison of studies difficult [17]. Only 3 studies to date have included older adults with significant co-morbidities [8], [12], [14], who are at increased risk of IPD.

**Table 1:** Summary of studies comparing conjugate (PCV7) and polysaccharides (23vPPV) pneumococcal vaccines in immunocompetent adults.

Study (ref)	Subjects	Vaccine status	Sample size	Vaccine schedule	Follow up interval (post-vaccine)	Serotypes tested	Assay used	Difference in immunogenicity
de Roux et al. [15]	≥70 years	Healthy elderly with no prior pneumococcal vaccine	219	PCV7/PCV7 or PCV7/23vPPV or 23vPPV/PCV7	Baseline, 1 month	4, 6B, 9V, 14, 18C, 19F, 23F	ELISA and OPA	PCV7 > 23vPPV except 19F (both assays). Hyporesponsiveness following 23vPPV-PCV7
Jackson et al. [16]	70–79 years	Healthy adults , no prior pneumococcal vaccine within 5 years	220	PCV7 (0.1, 0.5, 1 and 2 ml) or 23vPPV	Baseline, 1 month, 12 months	4, 6B, 9V, 14, 18C, 19F, 23F	ELISA and OPA	PCV7 (1 ml) > 23vPPV (0.5 ml)
Goldblatt et al. [13]	50–80 years	Healthy and no prior pneumococcal vaccine within 5 years	599	PCV7 or 23vPPV PCV7/PCV7 or PCV7/23vPPV	Baseline, 4–6 weeks, 12 months	4, 6B, 9V, 14, 18C, 19F, 23F	ELISA	PCV7 > 23vPPV (4, 9V, 23F). 23vPPV > PCV7 (19F). No advantage of 2^nd^ dose.
Lazarus et al. [9]	50–70 years	Healthy adults and no prior pneumococcal vaccine	348	23vPPV-PCV7-PCV7, PCV7-23vPPV-PCV7, PCV7-PCV7-23vPPV	Baseline, 4 weeks	4, 6B, 9V, 14, 18C, 19F, 23F	ELISA	PCV7 > 23vPPV (4, 9V, 18C, 23F). No advantage in priming of PCV7 with 1 or 2 doses of 23vPPV. Both 23vPPV-PCV7 and 23vPPV-PCV7-PCV7 < PCV7, except 19F.
Baxendale et al. [10]	52–74 years	Healthy adults with no prior pneumococcal vaccine within 5 years	37	PCV7 or 23vPPV	Baseline, 7 days, 28 days	4, 6B, 9V, 14, 18C, 19F, 23F	ELISA	No difference
Ridda et al. [11]	≥60 years	No prior pneumococcal vaccine, hospitalised frail elderly*	241	PCV7 followed by 23vPPV or 23vPPV	Baseline, 6 months	4, 6B, 18C, 19F	ELISA	No difference
Miernyk et al. [12]	55–70 years	Naïve, Alaskan natives	86	23vPPV alone or PCV7 followed by 23vPPV at 2 & 6 months	Baseline, 7–14 days, 6–10 weeks	1, 4, 6B, 14, 19F	ELISA and OPA	No difference
Dransfield et al. [8]	>40 years	Moderate/severe COPD patients with naïve or no prior Pneumococcal vaccine within 5 years)	181	PCV7 or 23vPPV	Baseline, 1 yr, 2 yrs	4, 6B, 9V, 14, 18C, 19F, 23F	ELISA and OPA	PCV7 > 23vPPV (4, 6B, 9V, 14, 18C, 23F) at both 1& 2 years.
Musher et al. [14]	Adults (average age >60 years)	Patients recovered from pneumococcal pneumonia (76% had prior pneumococcal vaccine)	81	PCV7/23vPPV or 23vPPV/PCV7	Baseline, 4–8 weeks, 6 months	4, 6B, 9V, 14, 18C, 19F, 23F	ELISA and OPA	At 4–8 wks: PCV7-23vPPV> 23vPPV-PCV7 At 6 months: no difference

*Preliminary report with in-house ELISA of sub-set of participants from this study.

Strain replacement, antibiotic resistance, poor immune responses and the continued ageing of the population pose an on-going challenge in the elderly [18]. The proportion of the population aged 65 years and over is growing, and is projected to increase to 25% by the middle of this century [19]. Vaccination against pneumococcal disease is one of the readily available preventive health strategies for the elderly. In this paper, we aimed to compare the immunogenicity of 23vPPV and PCV7 alone and following a dose of 23vPPV given to PCV recipients, in vaccine-naïve, hospitalized adults ≥60 years of age.

## Methods

### Ethics statement

The study was approved by the Human Research Ethics Committees (HREC) of Sydney West Area Health Service, The Children's Hospital at Westmead and the University of Sydney, as well as by the New South Wales Guardianship Tribunal. Each participant or her/his legal representative signed the written informed consent prior to enrolment.

### Study design and eligibility

We conducted a randomized, controlled clinical trial to compare PCV7 and 23vPPV in unvaccinated, hospitalised adults aged ≥60 years. Participants were recruited from the geriatric, cardiology, orthopaedic or rheumatology wards at a tertiary referral hospital in Sydney, Australia, which serves a population of 1,114,020 people [20]. We previously reported on a subset of the same hospitalised, frail elderly subjects using preliminary in-house ELISA testing only for four serotypes (4, 6B, 18C and 19F) [11]. This study reports on a larger number of subjects for 10 serotypes using standardized methods for both ELISA and OPA at the laboratories of Pfizer Vaccine R&D, Pearl River, NY 10965. The protocol for this trial and supporting Consort checklist are available as supporting information; see Protocol S1 and Checklist S1.

Eligible subjects were hospitalised adults aged ≥60 years who had never received pneumococcal vaccine. The patients' physicians were contacted to validate self-reported vaccination status. Participants were eligible if they were stable and able to give consent. We also obtained next-of-kin consent and Guardianship Board approval for patients who were demented or did not have capacity to consent.

**Figure 1:**
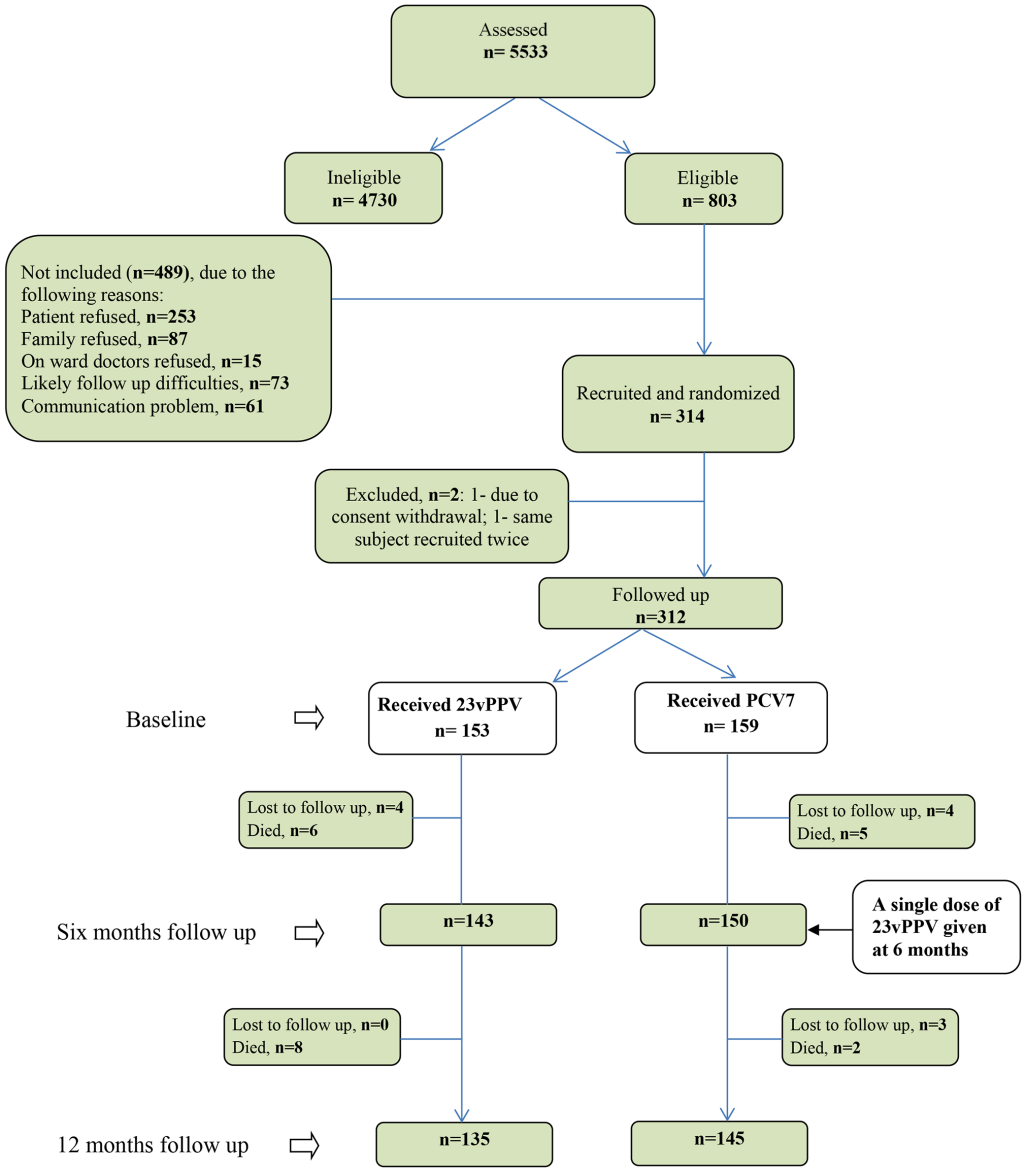
Flow Diagram of recruitment for the trial.

Using a randomized, controlled clinical trial design, subjects were recruited from May 2005 through December 2007, and the trial follow-up completed on February 2008. Participants were randomized by secure computerized randomization to receive either 23vPPV (control - standard recommended vaccine) or PCV7 (intervention) by a single injection in the deltoid muscle. Those who received PCV7 at baseline received a dose of 23vPPV 6 months later as shown in *Figure 1*. Vaccine was open label, but laboratory testing was blinded, given outcomes were immunological responses.

Serum samples were obtained from patients at baseline, six and 12 months after vaccination, and stored at −80°C until tested. The approving HREC did not approve collection of serum at 4 weeks post-vaccination, because of concerns of subjecting frail elderly patients to an additional blood collection. In light of the request to reduce the number of serum collections, it was felt that functionally, in terms of sustained response, 6 months was a more important measure.

The outcomes were antibody levels as measured by ELISA and OPA at 6 months (compared to baseline) and 12 months. Adverse events following vaccination were monitored on day 1 and day 7 after each vaccination and graded as mild, moderate or severe.

At the time the trial was funded by the National Health and Medical Research Council of Australia in 2004, and when it commenced in January 2005, the requirement for trial registration was not universally present. The trial was registered while in progress in 2007, at a time when universal registration was gaining wide acceptance, on the Australian Clinical Trials Registry # ACTRN 12607000387426 (http://www.anzctr.org.au/). The authors confirm that all on-going and related trials for this intervention are registered.

#### Vaccines

23vPPV (Merck Pneumovax) consists of a mixture of purified capsular polysaccharides from 23 serotypes of *S. pneumoniae*: 1, 2, 3, 4, 5, 6B, 7F, 8, 9N, 9V, 10, 11A, 12F, 14, 15B, 17F, 18C, 19F, 19A, 20, 22F, 23F, and 33F. The vaccine does not contain an adjuvant.

PCV7 (Wyeth/Pfizer Prevenar7), which is currently licensed for pediatric use, contains polysaccharides of pneumococcal serotypes 4, 6B, 9V, 14, 18C, 19F, and 23F. The polysaccharides are conjugated to the protein carrier CRM_197_. The vaccine contains aluminium phosphate as an adjuvant.

#### Measurement of frailty and comorbidity

Activities of daily living (ADL) and frailty were measured using the Barthel Index (BI) [21] and a modified Frailty Index (FI) [22], [23], respectively. The BI is a summary score of functional level. The Frailty Index has a list of 40 items, mostly comorbidities, and a total score is summed for each patient. A score of 0 is the least frail and a maximum of 40 indicates extreme frailty. High frailty reflects a high number of co-morbid conditions.

We also assessed cognition function by using the standard Mini-Mental Status Examination (MMSE) [24]. Our criteria required relative/guardian consent for a MMSE score of less than 20.

### Outcomes and measurement of pneumococcal antibodies

The primary endpoint was serological response to vaccines as measured by ELISA and OPA. OPA was done to measure functional antibody status, given there is no robust protective threshold value of ELISA for frail hospitalized elderly people. Laboratory testing for OPA activity and ELISA anti-PS IgG antibody concentrations were conducted at the laboratories of Pfizer Vaccine R&D, Pearl River, NY 10965.

#### ELISA anti-PS IgG antibody concentrations

Anti-pneumococcal polysaccharide (PnPs) antibodies against vaccine serotypes 3, 4, 6A, 6B, 9V, 14, 18C, 19A, 19F, 23F were measured by ELISA using both C-PS and 22F serotype capsular PS absorption, as described previously [25].

#### Opsonophagocytic assay

The opsonic activities of the samples were evaluated by OPA for serotypes 4, 6A, 6B, 9V, 14, 18C, 19A, 19F, 23F as previously described [26]. Serotype 3 was unable to be tested due to insufficient volumes of sera. The limit of detection for each of the OPAs was a titre equal to 1∶8.

### Sample size calculations and statistical evaluation

A sample size of 132 subjects per arm was required to test the difference in reactogenicity and response rate between groups with the estimated standard deviation of 0.55 for the change in log10-22FA levels within any group. We recruited 153 in 23vPPV arm and 159 in PCV7-23vPPV arm to allow for loss to follow up.

Comparisons of geometric means of the antibody concentrations (GMC) of ELISA (µg/mL) and geometric mean titres (GMT) of OPA (titer^−1^) at baseline, 6 months and 12 months were performed for pneumococcal serotypes 3, 4, 6A, 6B, 9V, 14, 18C, 19A, 19F, 23F, using unpaired 2-tailed *t*-test for parametric analyses and Wilcoxon rank sum test for non-parametric analyses, respectively. The 95% confidence intervals were constructed by back transformation of the confidence intervals for the mean of the log transformed assay results computed using the Student *t* distribution [27]. Reserve cumulative distribution (RCD) curves were used to evaluate the full spectrum of the immune response (ELISA GMC, IgG, µg/mL), and p-value was calculated with non-parametric log-rank test to compare RCD curves between PCV7 and 23vPPV arms (*Appendix S1*). We further analysed both ELISA and OPA responses to vaccination according to whether subjects had detectable OPA antibody at baseline. ELISA responses were also analysed by frailty and age group (over and under 75 years of age) to determine if age and frailty predicted response. A modified FI [22], [23] with the minimum score 0 (least frail) and the maximum 40 (most frail) was used to categorise subjects into low (FI≤10), moderate (11≤FI≤15) and severe (FI≥16) frailty groups. We compared the proportion of participants in each arm of the trial who experienced local or systemic adverse events as a result of the 1^st^ dose using a chi-squared test or Fisher's exact test. We also compared the proportions of participants who experienced adverse events who received PCV7 in their first dose and 23vPPV in their second dose using McNemar's test (exact if small cell sizes). Statistical analysis was performed with SAS 9.2 system and figures in this paper were produced using Matlab R2010b.

## Results

A total of 5533 subjects were assessed for eligibility, and 312 recruited (*Figure 1*). Of these, 153 received 23vPPV (23vPPV only group) and 159 received PCV7, followed by 23vPPV at six months (PCV7-23vPPV group). There were no significant differences at baseline between two vaccine groups as shown in *Table 2*. During the 12 month period of the study, 21 subjects died. The deaths were from unrelated causes, with no significant differences in the death rates between arms (*Table 2*). Over 40% of subjects had multiple co-morbidities (as measured by FI), and almost all subjects had at least one co-morbid condition. Almost half the subjects had a moderate or high level of dependency as measured by BI, and about 11% had cognitive impairment in 23vPPV group (*Table 2*).

**Table 2:** Comparison of patients' characteristics between two vaccine groups (n = 312).

*Patients' characteristics*	*Vaccine groups*		
	23vPPV (n = 153)	PCV7-23vPPV (n = 159)	p-value
**Age**			
Mean	69.97	70.38	p = 0.692
SD	8.94	9.33	
Median	66.63	66.46	
**Age group (Years)**			
60–64	55 (35.95%)	53 (33.33%)	p = 0.793
65–74	55 (35.95%)	63 (39.62%)	
75–100	43 (28.10%)	43 (27.04%)	
**Sex**			
Male	79 (51.63%)	87 (54.72%)	p = 0.585
Female	74 (48.37%)	72 (45.28%)	
**Frailty Index** *			
Low (1–10)	83 (54.25%)	99 (62.26%)	p = 0.338
Moderate (11–15)	44 (28.76%)	36 (22.64%)	
Severe (16–24)	26 (16.99%)	24 (15.09%)	
**Death during study period**	14 (9.15%)	7 (4.40%)	p = 0.094
**Mini-Mental State**			
Impaired (≤18)	17 (11.11%)	8 (5.03%)	p = 0.064
Borderline (19–22)	148(11.76%)	13 (8.18%)	
Normal (≥23)	118 (77.12%)	138 (86.79%)	
**Barthel index (disability scores)**			
Severe dependency (0–49)	11 (7.19%)	7 (4.40%)	p = 0.427
Moderate dependency (50–90)	68(44.44%)	66 (41.51%)	
Minimal dependency (91–100)	74 (48.37%)	86 (54.09%)	

*The Frailty index sums comorbid conditions.

### Antibody responses

#### IgG antibody measured by ELISA

*Table 3* shows the geometric mean concentrations (GMC) of IgG antibody measured by ELISA. There were no significant differences between arms at baseline. At six months, the GMC for all serotypes and both vaccines were significantly higher than baseline, with at least a twofold increase in all, except for serotypes 6A in both arms and 3 in the PCV7 group (serotype 3 is not present in PCV7). The response to 9V (common to both vaccines) was significantly higher in the PCV7 arm compared to the 23vPPV arm. The GMC ratio was close to 1.0 for other antibody comparisons except for serotypes 14, 19A and 3 (latter contained only in 23vPPV) (*Table 3*). At 12 months antibody levels in both arms were still higher than at baseline, particularly for serotypes 14, 18C, 19A, 19F and 23F. The GMC of IgG antibody for serotype 3 was now similar between arms (ratio 0.88, 95% CI 0.64 to 1.22) and the antibody levels of both 9V and 23F were significantly higher in the PCV7-23vPPV arm (ratios 1.57 and 1.46 respectively, *Table 3*).

**Table 3:** Comparison (t-test) of GMC (µg/mL) between 23vPPV and PCV7.

Sero-type	GMC (ELISA, µg/mL) at Baseline				GMC (ELISA, µg/mL) at 6 months				GMC (ELISA, µg/mL) at 12 months			
	23vPPV (n = 153)^b^	PCV7 (n = 159)^c^	Ratio(95%CI)^a^	p-value	23vPPV (n = 153)^d^	PCV7 (n = 159)^e^	Ratio(95%CI)^a^	p-value	23vPPV (n = 153)^f^	PCV7-23vPPV (n = 159)^g^	Ratio(95%CI)^a^	p-value
3	0.51 (143)	0.41(148)	0.81 (0.60–1.10)	0.18	**1.20 (141)**	**0.45 (142)**	**0.37 (0.27–0.51)**	**<0.01**	0.91 (130)	0.80 (141)	0.88 (0.64–1.22)	0.44
4	0.29 (149)	0.24 (157)	0.84 (0.60–1.17)	0.30	1.36 (141)	1.60 (149)	1.17 (0.81–1.71)	0.40	1.00 (133)	1.37 (145)	1.38 (0.96–1.96)	0.08
6A	1.70 (151)	1.66 (157)	0.98 (0.78–1.23)	0.85	2.79 (140)	2.71 (150)	0.97 (0.74–1.27)	0.84	2.27 (133)	2.51 (146)	1.11 (0.86–1.42)	0.44
6B	1.49 (149)	1.62 (157)	1.09 (0.84–1.40)	0.53	4.47 (141)	4.38 (148)	0.98 (0.70–1.37)	0.91	3.34 (129)	3.91 (145)	1.17 (0.86–1.60)	0.32
9V	1.00 (151)	1.02 (156)	1.02 (0.78–1.34)	0.88	**3.35** **(142)**	**4.70** **(147)**	**1.40 (1.01–1.96)**	**0.04**	**2.61** **(134)**	**4.11** **(146)**	**1.57 (1.16–2.14)**	**<0.01**
14	2.69 (151)	2.13 (157)	0.79 (0.57–1.10)	0.17	12.80 (142)	9.93 (149)	0.78 (0.52–1.17)	0.22	9.20 (134)	8.72 (145)	0.95 (0.63–1.43)	0.80
18C	0.99 (152)	0.90 (159)	0.91 (0.67–1.25)	0.57	6.30 (141)	6.18 (147)	0.98 (0.69–1.39)	0.92	4.68 (134)	4.94 (145)	1.06 (0.77–1.46)	0.74
19A	3.15 (153)	3.31 (159)	1.05 (0.83–1.33)	0.67	10.13 (142)	7.90 (148)	0.78 (0.59–1.04)	0.09	8.49 (134)	9.15 (146)	1.08 (0.81–1.44)	0.61
19F	0.51 (149)	1.10 (152)	0.91 (0.67–1.22)	0.52	3.27 (139)	3.24 (140)	0.99 (0.66–1.48)	0.96	2.72 (133)	3.51 (142)	1.29 (0.88–1.88)	0.19
23F	0.29 (150)	1.18 (157)	1.03 (0.79–1.34)	0.84	3.23 (139)	4.29 (145)	1.33 (0.93–1.89)	0.11	**2.59** **(133)**	**3.78** **(144)**	**1.46 (1.06–2.02)**	**0.02**

a Ratio of PCV7 to 23vPPV.

b The number of samples tested for each stereotype varied depending on volume of available sera and ranged from 143–153.

c The number of samples tested for each stereotype varied depending on volume of available sera and ranged from 148–157.

d The number of samples tested for each stereotype varied depending on volume of available sera and ranged from 139–142.

e The number of samples tested for each stereotype varied depending on volume of available sera and ranged from 140–150.

f The number of samples tested for each stereotype varied depending on volume of available sera and ranged from 129–134.

g The number of samples tested for each stereotype varied depending on volume of available sera and ranged from 141–146.

**Figure 2:**
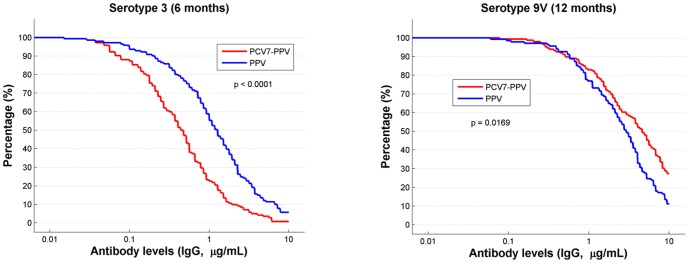
Reverse cumulative distribution (RCD) graphs for serotypes 3 at 6 months and serotype 9V at 12 months (based on ELISA IgG, µg/mL).

*Figure 2* illustrates the reverse cumulative distribution (RCD) curve of ELISA testing. For most serotypes, there was no difference in the curves (*Appendix S1*). The exceptions were serotypes 3 at 6 months and 9V at 12 months, as shown in *Figure 2*.

*Table 4* shows the GMC for IgG antibody compared in vaccine groups between subjects aged <75 years and >75 years. GMCs were not significantly different by age group for any serotype at baseline, but there was a uniform pattern of lower responses among the older age group post vaccination. At 6 months following a dose of 23vPPV, GMCs were significantly lower in those >75 years for serotypes 3, 6A, 6B, 9V, 14 and 18C. At 6 months, in both trial arms, subjects aged >75 years had significantly lower responses to serotypes 3, 6A, 6B and 9V. Following a dose of PCV7, responses were significantly lower in the >75 year age group for serotypes 19F and 23F. At 12 months following 23vPPV, GMCs had decreased in both age groups, but more in younger subjects who had reached higher post vaccination levels, and the only significant differences by age were for serotypes 6A, 14 and 18C. In the PCV7 group, 6 months following a dose of 23vPPV, there was a persistently and significantly lower GMC in subjects ≥75 years, compared to subjects <75 years, for serotypes 6A, 19F and 23F.

**Table 4:** Comparison of GMC (geometric mean concentration) ELISA, IgG, µg/mL (dichotomous analysis) between oldest age group (Age ≥75 yrs, n = 86) and other (Age <75 yrs, n = 226).

23vPPV (GMC, ELISA, IgG, µg/mL)										PCV7 (GMC, ELISA, IgG, µg/mL)								
*Serotype*	*Baseline*			*6 months*			*12 months*			*Baseline*			*6 months*			*12 months (PCV7-23vPPV)*		
	Age<75	Age≥75	p-value	Age<75	Age≥75	p-value	Age<75	Age≥75	p-value	Age<75	Age≥75	p-value	Age<75	Age≥75	p-value	Age<75	Age≥75	p-value
3	0.56	0.39	0.18	**1.37**	**0.80**	**0.04**	0.99	0.68	0.20	0.45	0.32	0.15	**0.53**	**0.28**	**<0.01**	0.85	0.66	0.31
4	0.27	0.35	0.33	1.54	0.95	0.10	1.11	0.70	0.14	0.22	0.31	0.17	1.75	1.23	0.27	1.42	1.24	0.65
6A	1.73	1.60	0.67	**3.11**	**2.00**	**<0.05**	2.52	1.61	0.04	1.77	1.37	0.15	**3.21**	**1.67**	**<0.01**	**2.79**	**1.79**	**0.03**
6B	1.58	1.30	0.34	**5.21**	**2.85**	**0.02**	3.67	2.46	0.12	1.69	1.44	0.44	**5.16**	**2.78**	**0.03**	4.19	3.13	0.28
9V	1.05	0.86	0.35	**3.92**	**2.11**	**0.01**	2.88	1.89	0.08	1.00	1.06	0.81	**5.57**	**2.89**	**0.03**	4.40	3.31	0.30
14	2.76	2.50	0.70	**16.22**	**6.37**	**<0.01**	11.41	4.50	0.01	2.24	1.86	0.49	11.53	6.41	0.08	9.36	6.91	0.35
18C	1.01	0.93	0.75	**7.73**	**3.39**	**<0.01**	5.57	2.62	<0.01	0.84	1.09	0.30	7.02	4.30	0.11	5.04	4.66	0.78
19A	3.21	2.99	0.70	11.31	7.32	0.06	8.98	7.04	0.34	3.37	3.15	0.72	8.71	5.89	0.10	9.15	9.14	0.99
19F	1.25	1.13	0.69	3.73	2.26	0.13	2.94	2.11	0.34	1.18	0.92	0.30	**4.07**	**1.68**	**<0.01**	**4.05**	**2.21**	**0.04**
23F	1.13	1.19	0.83	3.47	2.61	0.26	2.78	2.04	0.23	1.25	1.00	0.30	**5.38**	**2.17**	**<0.01**	**4.46**	**2.26**	**0.02**

For 23vPPV group, both younger and older increased substantially post vaccine for all serotypes and at least 2 fold rise except for 6A – not in 23vPPV- which increased by 1.8 and 1.25 for <75 yrs and ≥75 yrs respectively. Types 14 and 18C showed greatest increment of 5.9 and 7.6 fold respectively for <75 yrs but fold increase was sig less for ≥75 yrs of 2.6 and 3.6 respectively.

For PCV7 group, also lower responses among ≥75 yrs for 7v types except 4 (also 23vPPV) which was particularly marked for 19F and 23F where there was not sig difference by age for 23v. Notably, GMCs among ≥75 yrs group were not higher 6 months post PCV7 compared with 23vPPV – in fact v similar 4 (0.95 vs 1.23), 6B (2.85 vs 2.78), 9V (2.11 vs 2.89), 14 (6.37 vs 6.41) 18C (3.39 vs 4.30), 19F (2.26 vs 1.68) and 23F (2.61 vs 2.17) – note 9V difference sig for overall group with ratio of 1.40 and this ratio is same for ≥75 yrs - 1.4. For other non 7v types, there are related types (6A and 19A) and one non 7v type (3). Sig ratio for 3, marginal sig (0.08) for 19A and non sig for 6A. Lower responses among ≥75 yrs for all serotypes for both vaccines types.

#### Opsonophagocytic antibody levels (OPA)

The geometric mean titres of OPA responses for both vaccines are summarized in *Table 5*. There were no significant differences in the ratio of OPA GMT between 23vPPV and PCV7 at baseline, but variability was greater and for one serotype (19F) the baseline GMT in the PCV7 group was significantly lower. There was an increase of more than twofold above baseline at 6 months, with levels remaining higher than baseline for both groups at 12 months. For PCV7 recipients, at 6 months there was a significantly higher GMT for serotypes 9V and 23F, with both remaining significantly higher than for 23vPPV recipients at 12 months (6 months following the 23vPPV dose). Among 23vPPV recipients at 6 months, OPA GMTs were significantly higher against serotypes 19F (common to both vaccines) and 19A (present only in the 23vPPV vaccine). In general, levels fell by 12 months post a single dose of 23vPPV to a modest extent, but usually remained more than twofold higher than baseline, except 19F and 23F. The GMC ratio of OPA between trial arms was significantly higher at 12 months for PCV7-23vPPV for serotypes 6A (not present in either vaccine), 9V, 18C and 23F. OPA levels for these serotypes increased moderately from the 6 month level, whereas waning of immunity was seen for the 23vPPV only arm. Among the PCV7/23vPPV group at 12 months, 6 months post the 23vPPV dose, OPA GMTs were no longer reduced for 19A or 19F compared to the 23vPPV group.

**Table 5:** Comparison (t-test) between 23vPPV and PCV7 for OPA in GMT (titre^−1^).

OPA GMT (titre^−1^) at baseline					OPA GMT at 6 months				OPA GMT at 12 months			
Serotype	23vPPV(n = 153)^b^	PCV7(n = 159)^c^	Ratio(95%CI)^a^	p-value	23vPPV(n = 153)^d^	PCV7(n = 159)^e^	Ratio(95%CI)^a^	p-value	23vPPV(n = 153)^f^	PCV7-23vPPV(n = 159)^g^	Ratio(95%CI)^a^	p-value
3	-	-	-	-	-	-	-	-	-	-	-	-
4	66 (84)	39 (84)	0.59 (0.31–1.11)	0.10	410 (57)	387 (60)	0.94 (0.39–2.25)	0.61	332 (76)	434 (84)	1.31 (0.68–2.53)	0.57
6A	84 (81)	70 (91)	0.83 (0.50–1.39)	0.47	259 (67)	215 (88)	0.83 (0.45–1.55)	0.72	**168 (109)**	**277 (120)**	**1.66 (1.02–2.69)**	**0.02**
6B	139 (59)	129 (57)	0.92 (0.44–1.92)	0.83	421 (77)	457 (93)	1.09 (0.60–1.96)	0.60	308 (91)	394 (97)	1.28 (0.74–2.21)	0.35
9V	338 (89)	281 (93)	0.83 (0.61–1.13)	0.32	**350 (82)**	**505 (103)**	**1.45 (1.03–2.04)**	**<0.05**	**364 (94)**	**640 (99)**	**1.76 (1.24–2.49)**	**<0.01**
14	164 (103)	148 (99)	0.90 (0.52–1.55)	0.65	516 (83)	371 (94)	0.72 (0.40–1.28)	0.21	418 (116)	594 (119)	1.42 (0.88–2.28)	0.13
18C	83 (97)	53 (85)	0.64 (0.37–1.10)	0.08	683 (96)	479 (114)	0.70 (0.42–1.17)	0.39	**336 (107)**	**694 (118)**	**2.06 (1.27–3.36)**	**<0.01**
19A	52 (84)	36 (86)	0.70 (0.41–1.20)	0.24	**253 (73)**	**88 (94)**	**0.35 (0.20–0.60)**	**<0.01**	167 (106)	173 (127)	1.04 (0.67–1.61)	0.68
19F	81 (79)	48 (81)	0.59 (0.36–0.97)	<0.05	**168 (93)**	**99 (111)**	**0.59 (0.37–0.93)**	**0.03**	144 (103)	133 (116)	0.92 (0.59–1.43)	0.68
23F	24 (82)	22 (72)	0.91 (0.48–1.74)	0.93	**57 (76)**	**114 (99)**	**2.00 (1.01–3.99)**	**<0.05**	**44 (108)**	**120 (120)**	**2.71 (1.50–4.88)**	**<0.01**

a Ratio of PCV7 to 23vPPV.

b The number of samples tested for each stereotype varied depending on volume of available sera and ranged from 59–103.

c The number of samples tested for each stereotype varied depending on volume of available sera and ranged from 57–99.

d The number of samples tested for each stereotype varied depending on volume of available sera and ranged from 57–96.

e The number of samples tested for each stereotype varied depending on volume of available sera and ranged from 60–103.

f The number of samples tested for each stereotype varied depending on volume of available sera and ranged from 76–116.

g The number of samples tested for each stereotype varied depending on volume of available sera and ranged from 84–127.

Sig difference identified by ELISA for 9V also identified by OPA and persisted to 12 months. Also, sig higher OPA for 23F in PCV7 group at 6 months which persisted at 12 months. Significantly higher OPA at 6 months for 23vPPV group for 19A and 19F – more marked for 19A - which was abolished at 12 months' time point (6 months post 23vPPV booster).

### Impact of frailty on immune response

**Figure 3:**
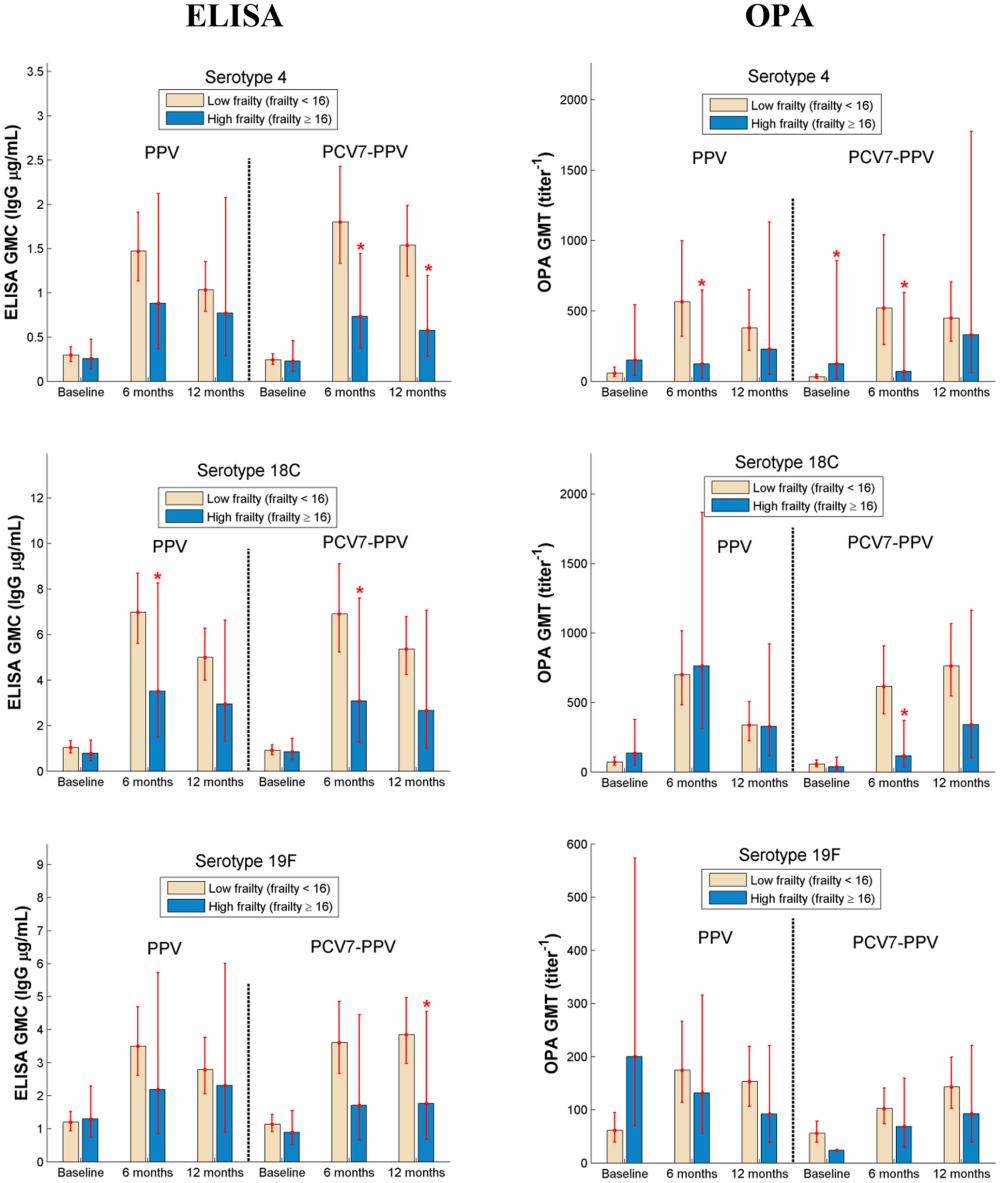
Comparison of immune responses (GMC of ELISA, IgG, µg/mL, GMT of OPA, titre^−1^) between low (frailty <16) and high (frailty ≥16) frailty groups. (* Significant difference between low frailty and high frailty for *t*-test, p≤0.05).

Subjects with high frailty had lower antibody levels post vaccination for all serotypes, as measured by both ELISA and OPA. *Figure 3* shows the 3 serotypes (4, 18C and 19F) where high frailty subjects had a significantly lower antibody response. Notably, there was no evidence of a superior OPA response at 6 months post vaccination for high frailty recipients of PCV7 compared with those who received 23vPPV, and for serotype 18C, 23vPPV recipients had better responses at 6 months.

### Impact of pre-vaccination immunity status on immune responses to PPV and PCV7

**Figure 4:**
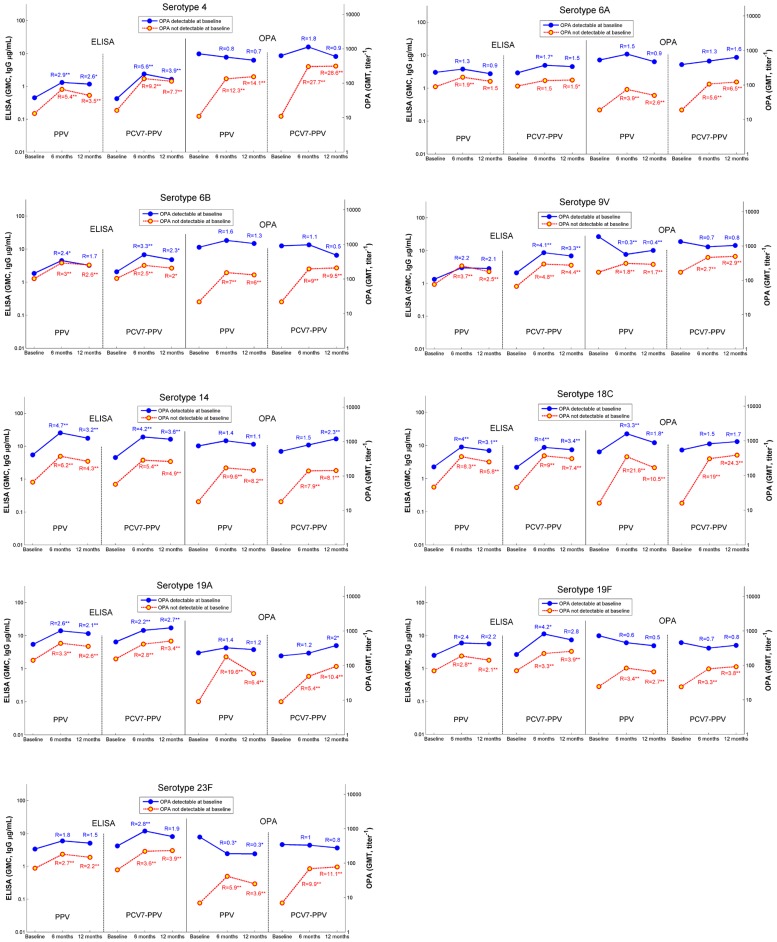
ELISA (IgG, µg/mL) and OPA titres (titre^−1^) by time point based on baseline titre (i.e., detectable and not detectable) for each of 9 serotypes: 4, 6A, 6B, 9V, 14, 18C, 19A, 19F and 23F (R = ratio to baseline; *significant difference from baseline, p≤0.05; **highly significant difference from baseline, p≤0.01).

Depending on serotype, between 28% (serotype 23F) and 60% (serotype 14) of subjects had detectable OPA (i.e., titre ≥1∶8) at baseline (DOB). Subjects with high frailty were significantly less likely to have DOB for serotypes 4, 9V and 19A (data not shown). Age alone was less likely to predict DOB than frailty, with OPA responses in subjects aged >75 years significantly lower only to serotype 4 (data not shown). We compared post vaccine responses between trial arms among subjects who had DOB and undetectable OPA antibody at baseline (UOB). All results are displayed in *Figure 4*. The magnitude of response to both vaccines is much greater among subjects with UOB. However, the absolute level of the antibody post vaccination remained lower in the UOB group for all serotypes and both vaccines at 6 and 12 months. ELISA responses to serotypes 4, 14, 18C, and 19A increased significantly in both the DOB and UOB groups. In both vaccination groups, OPA titres decreased slightly in those with DOB for serotypes 4, 9V, 19F and 23F (statistically significant for 9V and 23F among 23vPPV recipients).

### Adverse events (AE)

The most common adverse events in both vaccine groups after a single dose of either vaccine were pain and redness at the injection site. There were no significant differences in adverse events between two groups after the first dose. However, a booster dose of 23vPPV following an initial dose of PCV7 showed significant increase in the incidence of local adverse events compared with the first doses of either vaccine as shown in *Table 6*. We also found that for all serotypes except 14, subjects with pain at the injection site had significantly higher ELISA antibodies at 6 months (data not shown). No serious vaccine-related adverse events were reported in either study arm.

**Table 6:** Adverse events.

23vPPV		PCV7-23vPPV			
Adverse events (AE)	*1^st^ dose (23vPPV)*	*1^st^ dose (PCV7)*	p-value*	*2^nd^ dose (23vPPV)*	p-value**
Local AE	26.7% (40/150)	19.6% (31/158)	0.14	20.7% (31/150) versus 34.0% (51/150)	0.0065
Systemic AE	2.0% (3/150)	0.6% (1/158)	0.36	0.7% (1/150) versus 4.0% (6/150)	0.125

*compared with 1^st^ dose (23vPPV),

**compared with 1^st^ dose (PCV7).

## Discussion

This is the largest study of hospitalized elderly patients, for the first time including patients with severe dependency, cognitive impairment (including dementia), and high frailty, who are generally excluded from clinical trials [28]. We demonstrated superior responses by OPA to a single dose of PCV7 over 23vPPV for serotypes 9Vand 23F, but not for other serotypes; and a single dose of 23vPPV elicited a higher OPA response than PCV7 for serotypes 19A and 19F (Table 5). A schedule of PCV7 followed by a dose of 23vPPV six months later, results in sustained antibody responses to serotypes common in both vaccines (9V, 18C and 23F), as well as against serotype 6A (not present in either vaccine). Waning of OPA antibodies was seen in the 23vPPV-only arm at 12 months, but this waning did not occur in the PCV7-23vPPV arm, and in fact a modest increase in antibodies was seen for some serotypes. Given waning immunity in the elderly [18], scheduling of 23vPPv following PCV7 should be explored further. The priming effect of PCV7 followed by 23vPPV in our study contrasts with a previous study in healthy adults aged 50–70 years [9], which showed no priming benefit of PCV7 either with one or two doses prior to 23vPPV. However, these were healthy adults recruited from general practice, and not hospitalized, frail elderly as in our study. Two studies have reported a similar priming effect of PCV7 followed by 23vPPV, however these antibody responses were short-term only [14], [15]. Follow up studies are needed to determine immunity beyond 12 months, as well as persistence of immunity following PCV7 alone.

Increasing frailty and age both predicted poorer responses to the vaccines. Interestingly, frailty more than age better distinguished low baseline immunity as measured by undetectable OPA. An assessment of frailty should have a role in vaccination of adults for more effective use of pneumococcal vaccine. Furthermore, the relative response to vaccination is greater for people with low baseline immunity. This is a novel finding, not previously described, and has potential implications; as such individuals are at greatest of developing invasive pneumococcal disease. Our study and previous studies [14], [15] have found that the final level of antibody response achieved is higher in people with detectable levels of antibody at baseline. Whilst this indicates the capacity for more robust immune response in people with better baseline immunity, our analysis of undetectable OPA at baseline adds further understanding to this subject and challenges the view that the frail elderly do not respond to vaccination [18], [29]. We believe this finding highlights the value of vaccinating the frail elderly, despite weaker immunity, because even if the final level of antibody achieved is not as high as in less frail people, they may still mount a protective response. Our previous work has shown that demented frail elderly and those aged >80 years are significantly less likely to be given pneumococcal vaccine than younger, non-demented patients [30]. This suggests that vaccination providers make judgments to withhold vaccine based on age and frailty. Our findings show this is not justified, as such people as can mount a potentially valuable immunological response.

Our study has some limitations. First, the study was not blinded, but laboratory testing was, and since the primary outcome was antibody levels, this should not have resulted in any bias. Secondly, whilst serological endpoints may indicate clinical protection, they are only a proxy for clinical protection. The strengths of this study include the use of OPA, as well as the measurement of frailty. We previously reported on a subset of the same hospitalized, frail elderly subjects using preliminary in-house ELISA testing only for four serotypes [11]. This study reports on a larger number subjects for a greater number of serotypes using both ELISA and OPA assays tested at the Pfizer laboratories, and unlike the preliminary report, demonstrated some differences between the two vaccination schedules. This may be due to larger sample size, use of OPA testing and use of a standardized ELISA method. The use of OPA in this study clearly enabled better comparison between the vaccine schedules, but we also showed a good correlation between OPA and ELISA for most serotypes. We were also able to show that classifying people by baseline detectable and undetectable OPA is helpful in predicting the magnitude of responses to vaccination. It is also a strength that we were able to include a unique study population who have not been included in previous studies – our subjects were affected by comorbidities (over 40% the study population), dementia, dependency, polypharmacy and higher mortality, which makes such a trial inherently complex and difficult to conduct.

In recent years, 13-valent pneumococcal conjugate vaccine (PCV13) has replaced PCV7 for infant immunization in many developed countries, and is being trialled in adults aged ≥65 years with efficacy endpoints against pneumonia [31]. Regardless of the ultimate clinical value of PCV7 in adults, the proof of principle in this study should be applicable to other conjugate vaccines and the benefit of a schedule of 13-valent PCV followed by 23vPPV in frail elderly people should be explored and compared with 13-valent PCV alone.

An increased incidence of IPD caused by replacement strains has been reported in many countries [32]–[34], notably serotype 19A. Interestingly, 23vPPV, either alone or following PCV7, resulted in a good immune response to 19A, highlighting a benefit of this vaccine. The T-cell independent response elicited by polysaccharide vaccines in theory should not produce as good an immune response as conjugated vaccines, yet the studies to date have not shown an overwhelming advantage of conjugate vaccines in the elderly [17].

With a rapidly ageing population, an evidence base in the frail elderly and their response to vaccines is greatly needed. There have been conflicting results between the few published studies in the elderly about choice between pneumococcal polysaccharide and conjugate vaccines, or combined schedules of both vaccines in most populations who most need them [7], [9], [11], [15], [35]–[37]. The unique contribution of our study is the inclusion of hospitalized frail elderly patients, including patients with dementia and high levels of comorbidity, who are generally excluded from clinical trials, yet who suffer the highest burden of pneumococcal disease [28]. Continued aging of the population and an increasing proportion of frail elderly will place higher demands on healthcare system, particularly in industrialized countries. Better prevention of pneumococcal disease in the elderly has the potential for significant cost savings in healthcare.

In summary, a single dose of PCV7 is more immunogenic for two serotypes (9V and 23F), but a single dose of 23vPPV is more immunogenic for serotype 19F. Frailty better indicates baseline immunity than age, age and frailty both predict responses, but contrary to long-held beliefs, people with low baseline immunity do mount an immune response to vaccination. A schedule of conjugate followed by polysaccharide vaccines results in sustained antibody levels at 12 months in the elderly, who are otherwise subject to waning of immunity. Waning of immunity is a universal challenge that accompanies ageing, so strategies that prevent waning are valuable. Longer-term follow up data on the persistence of immunity and clinical protection following pneumococcal vaccination would be valuable to inform vaccination policy for the elderly.

## Supporting Information

Appendix S1 (DOCX)

Protocol S1 (PDF)

Checklist S1 (DOCX)
