# Supplementary material for: A Randomized Clinical Trial of the Immunogenicity of 7-Valent Pneumococcal Conjugate Vaccine Compared to 23-Valent Polysaccharide Vaccine in Frail, Hospitalized Elderly
Source: PLoS One. 2014 Apr 23;9(4):e94578. doi: 10.1371/journal.pone.0094578 (PMC3997415; doi:10.1371/journal.pone.0094578)
Supplement: Appendix S1 — (DOCX) [file pone.0094578.s001.docx]

**APPENDIX**

**Figure 1. Reverse cumulative distribution (RCD) curves for individual vaccine: serotypes 3, 4, 6A, 6B, 9V, 14, 18C, 19A, 19F and 23F at baseline, 6 months and 12 months (based on ELISA IgG, µg/mL), in comparison between PPV and PCV7-PPV, using log-rank test.**

| 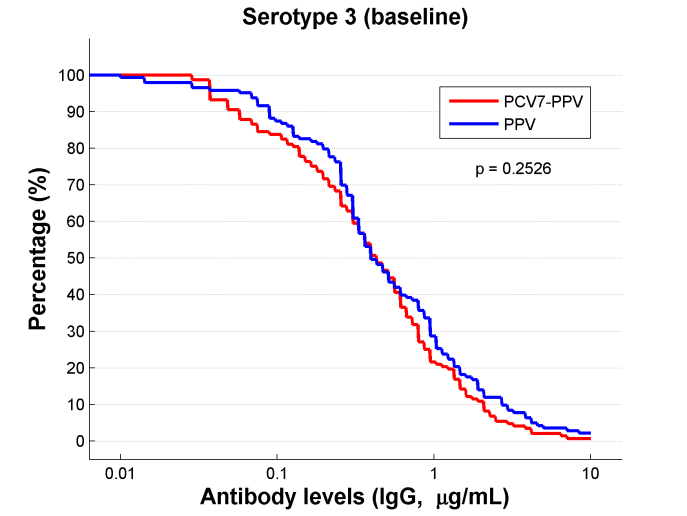 | 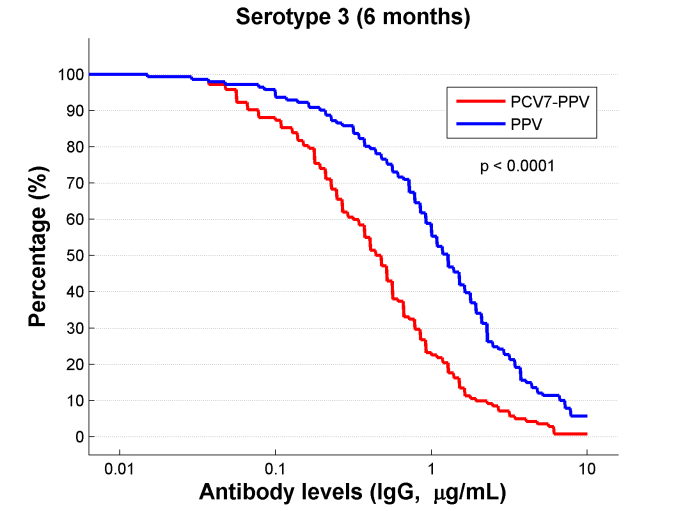 | 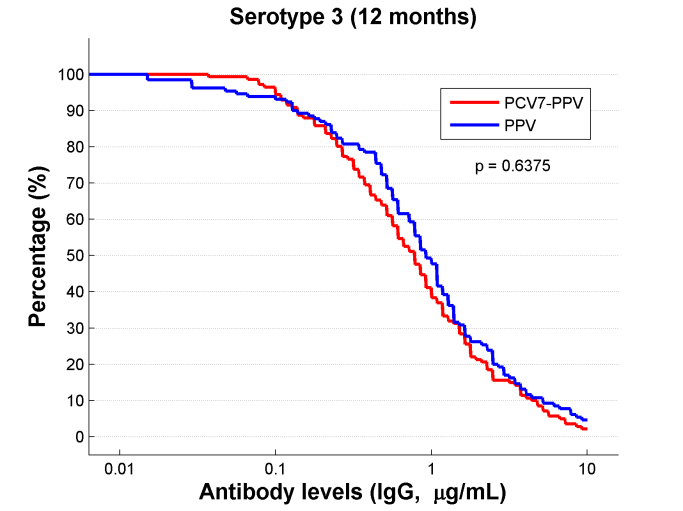 |
| --- | --- | --- |
| 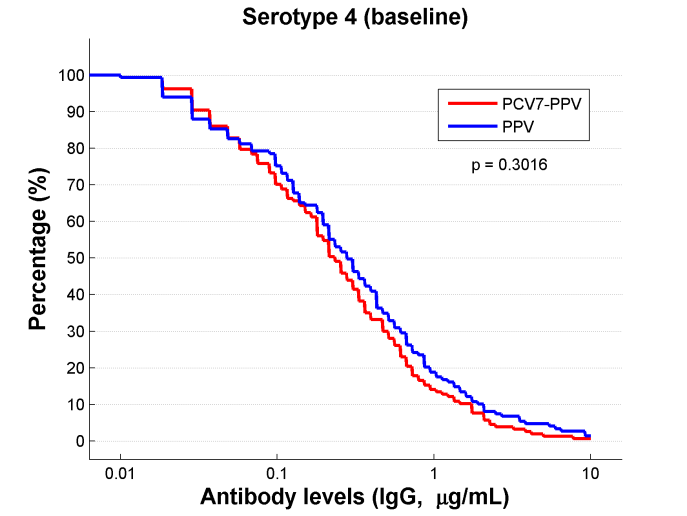 | 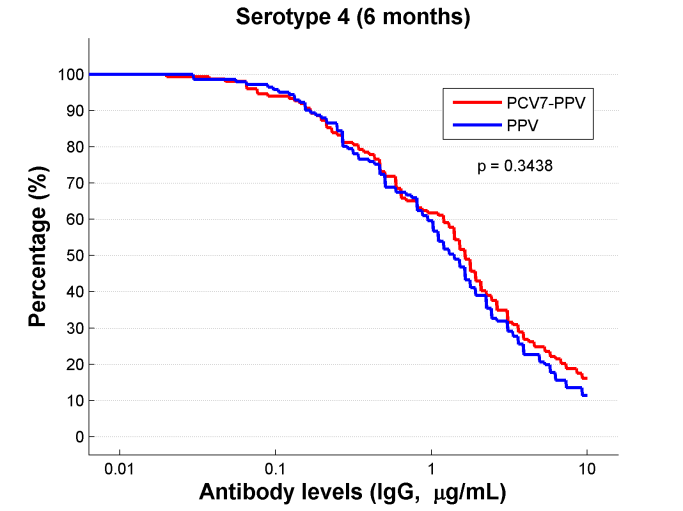 | 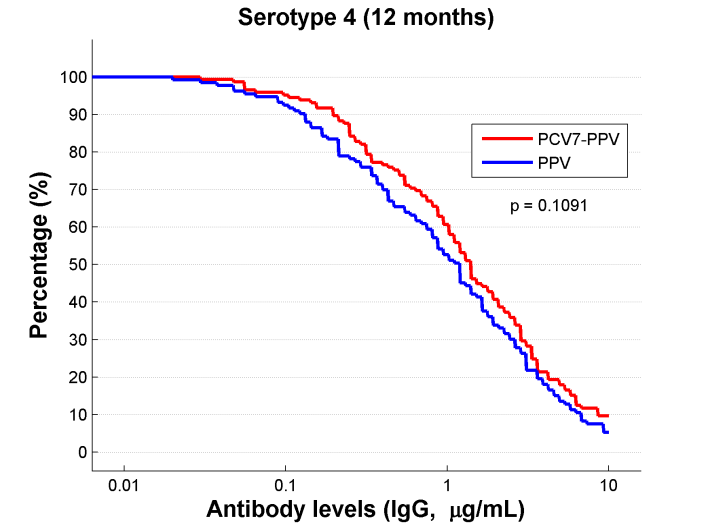 |
| 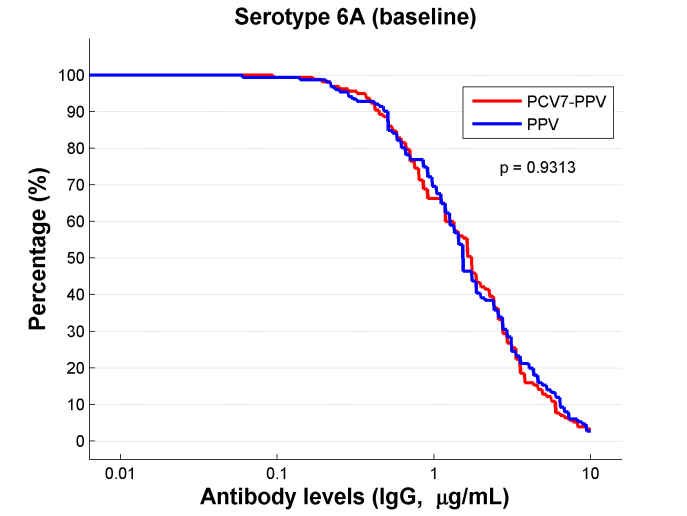 | 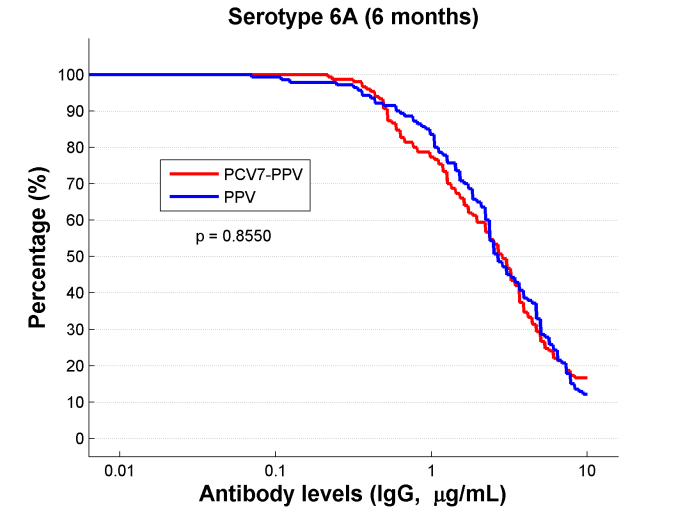 | 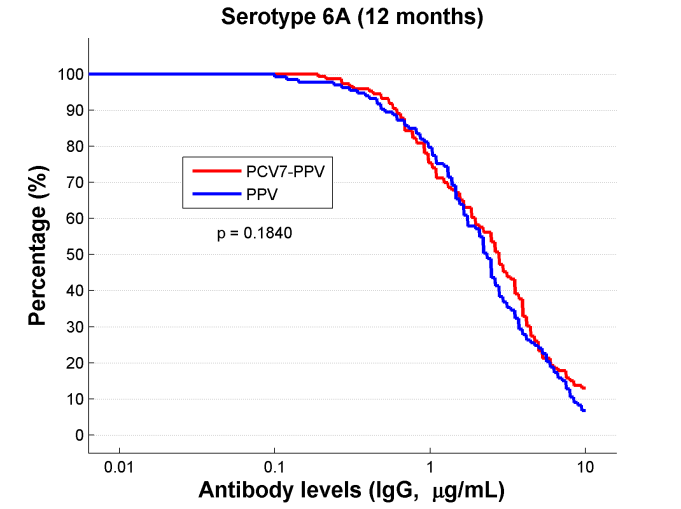 |
| 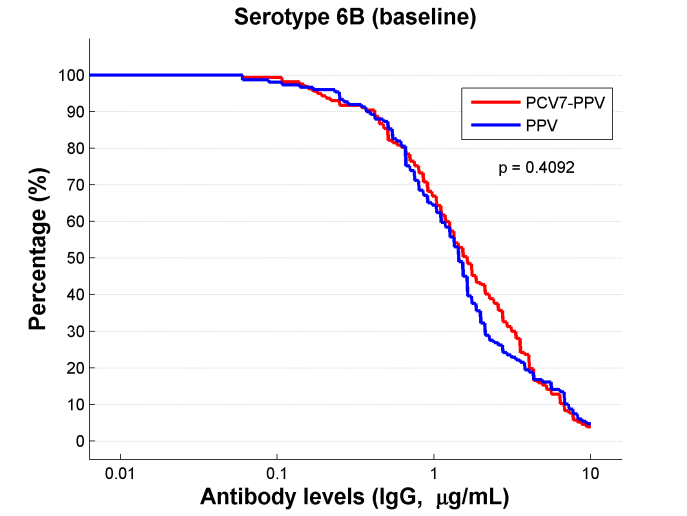 | 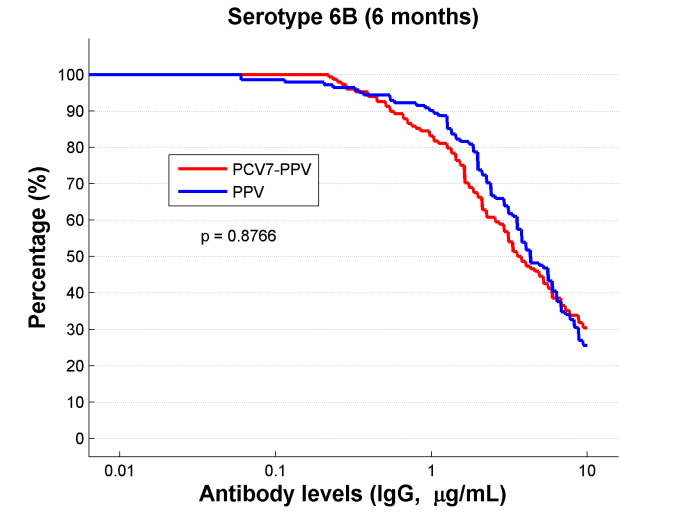 | 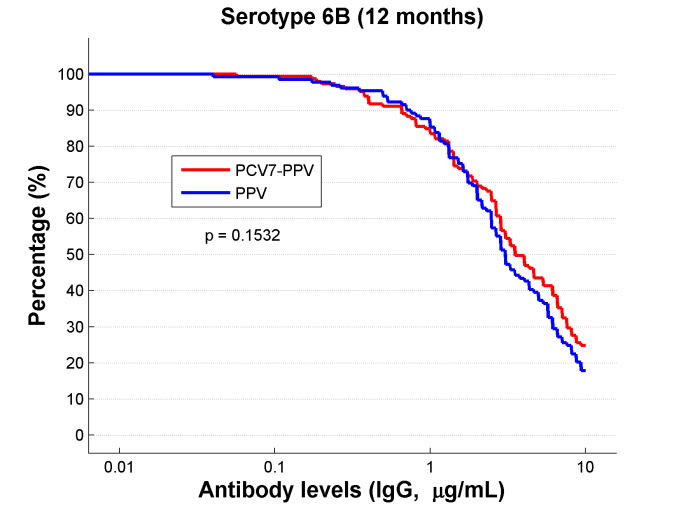 |
| 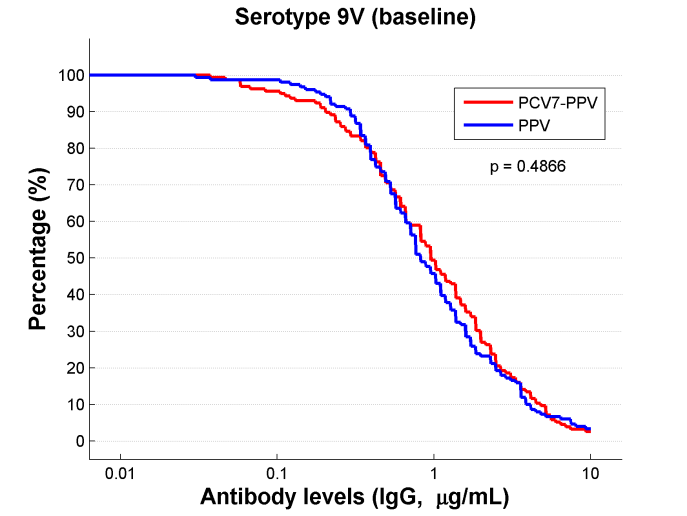 | 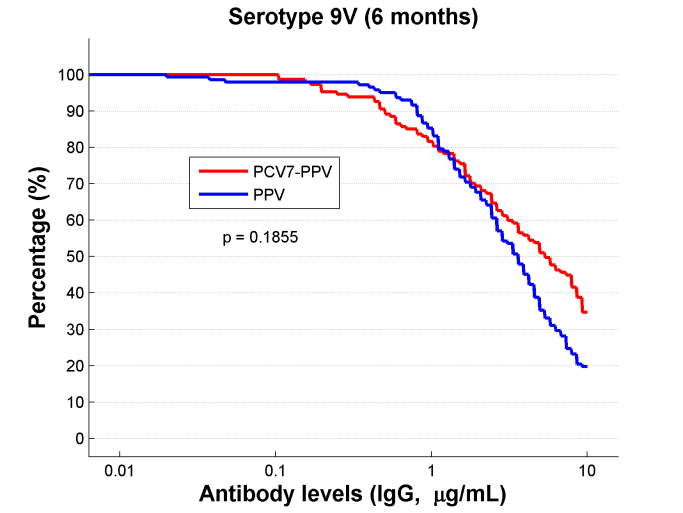 | 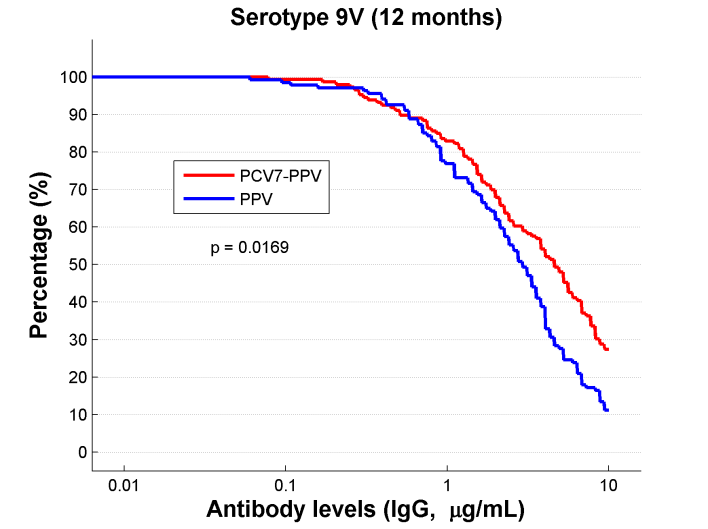 |
| 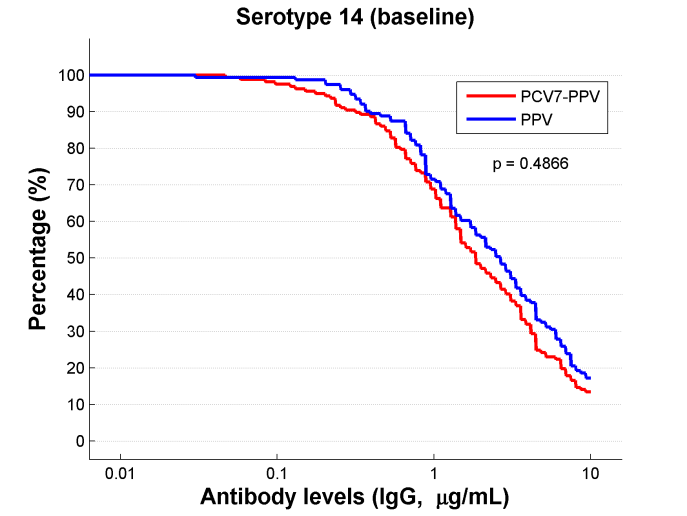 | 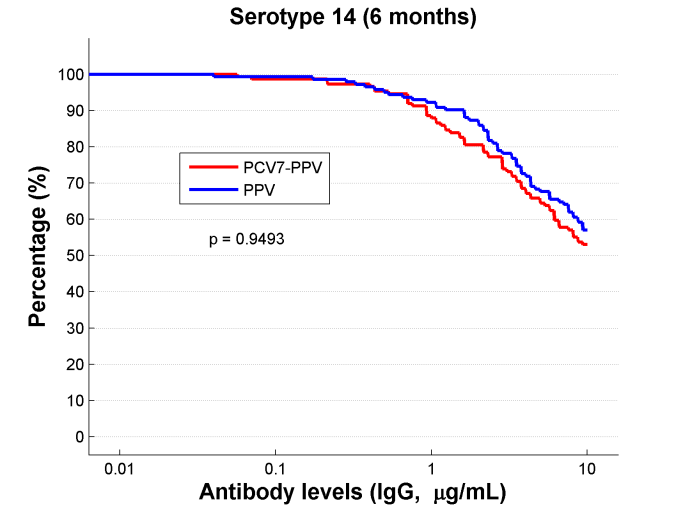 | 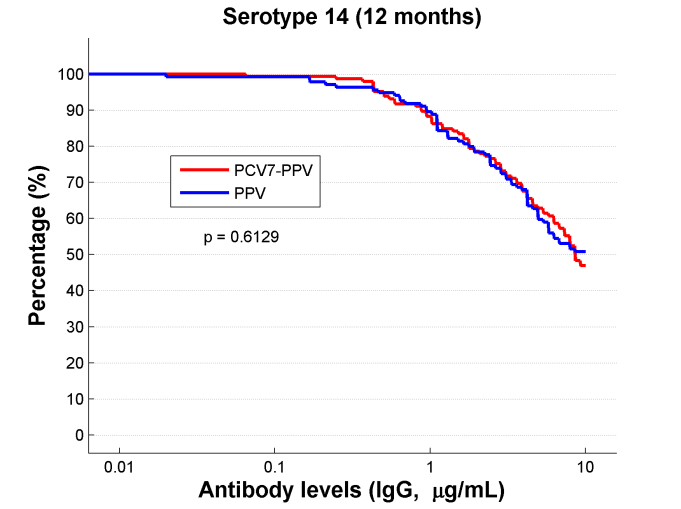 |
| 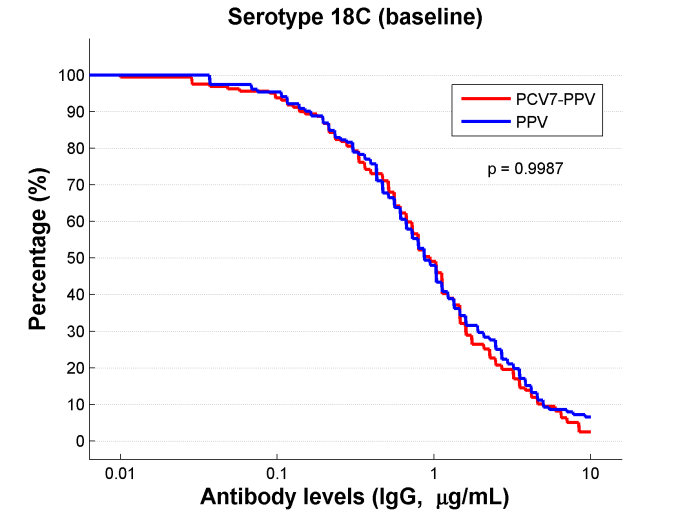 | 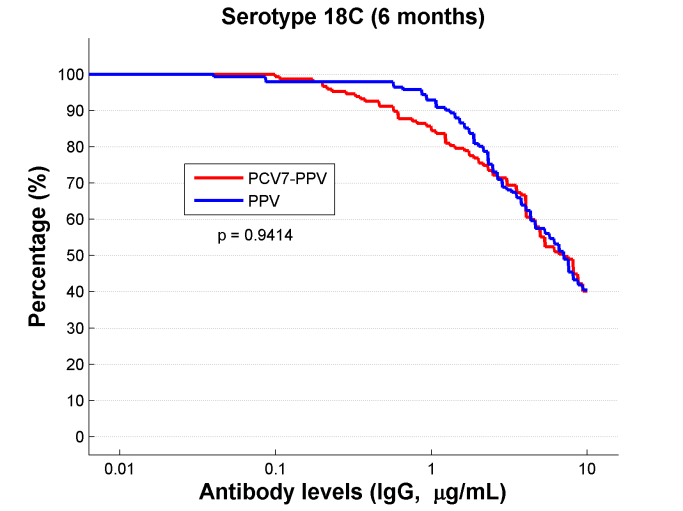 | 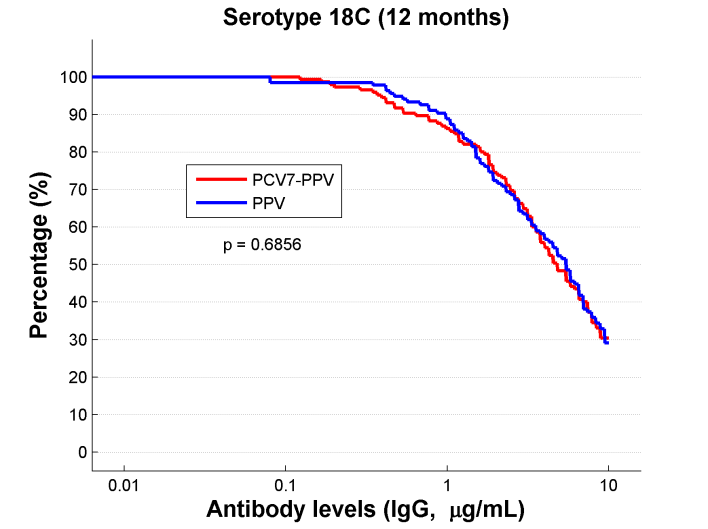 |
| 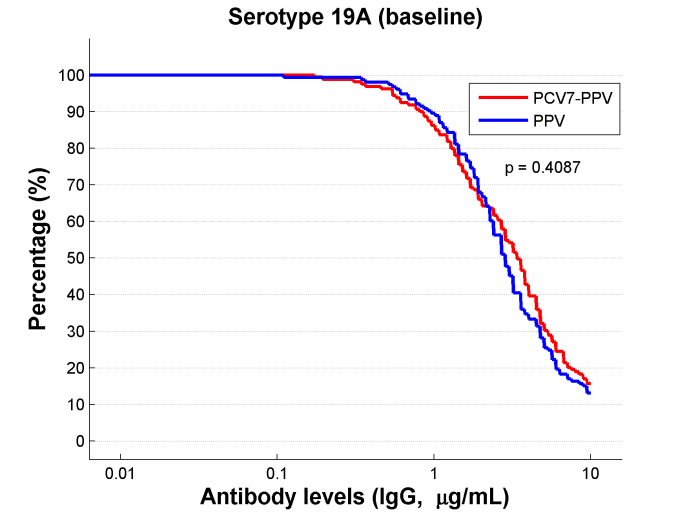 | 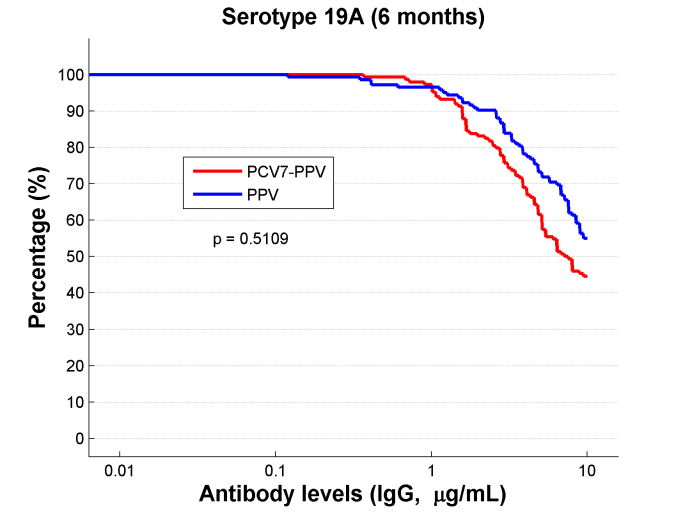 | 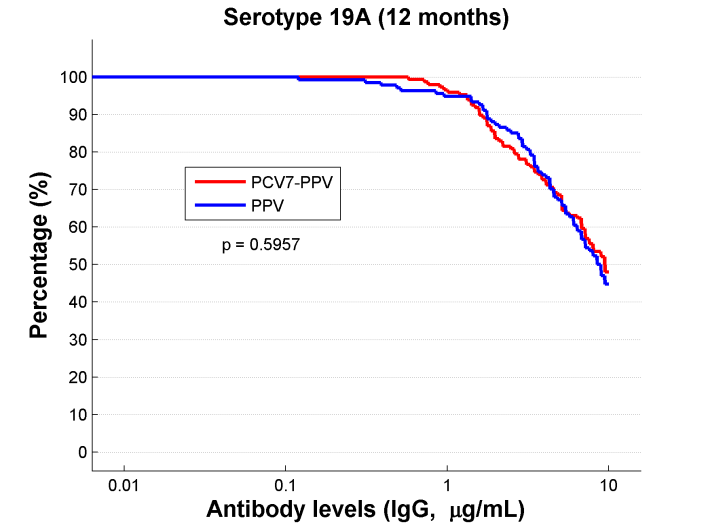 |
| 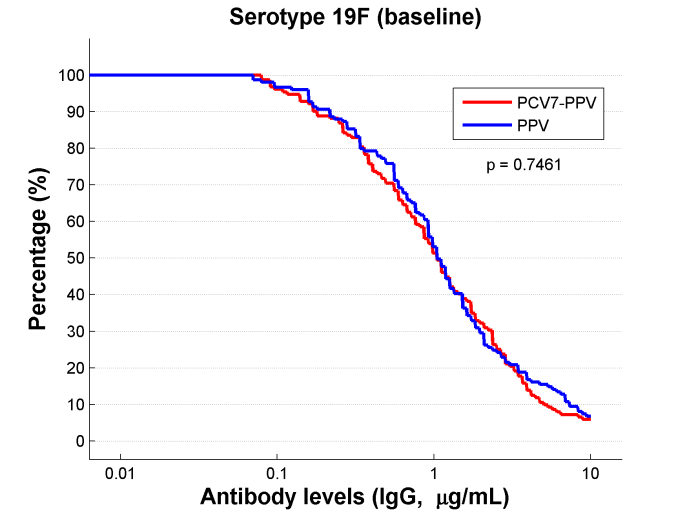 | 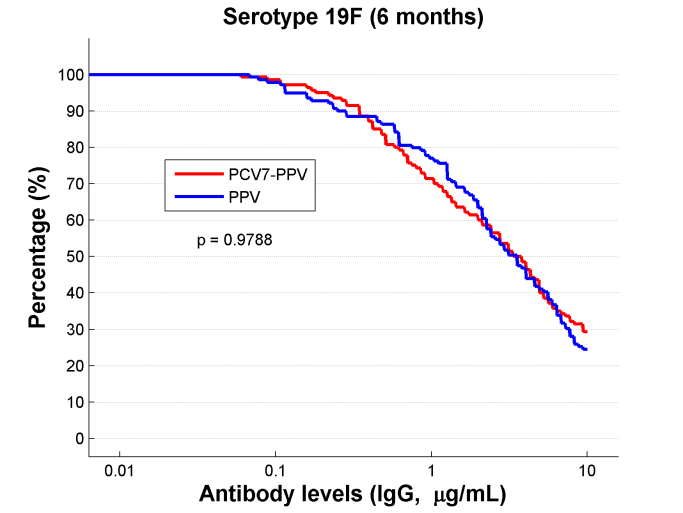 | 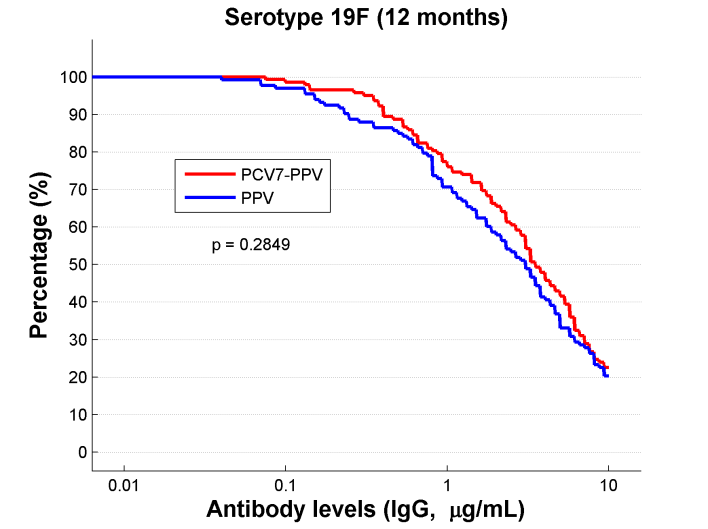 |
| 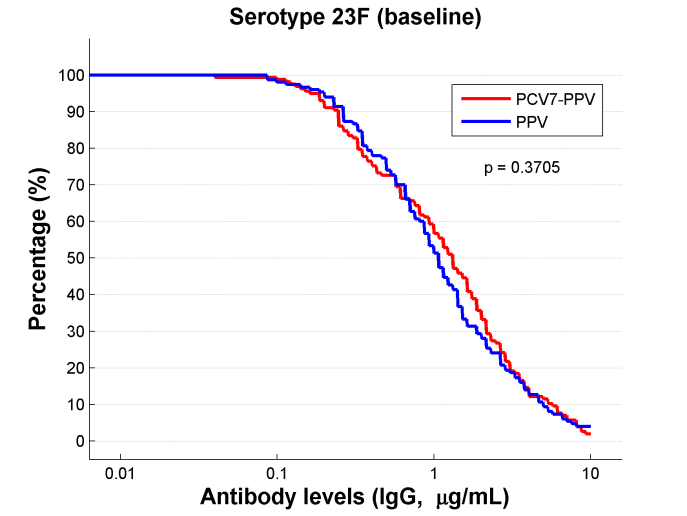 | 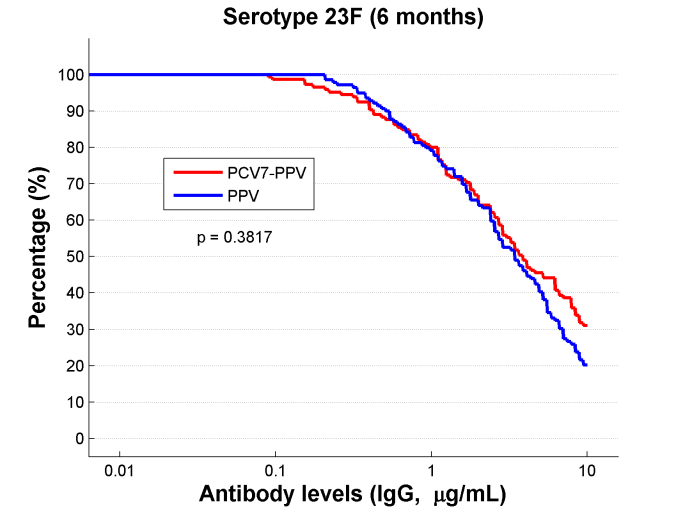 | 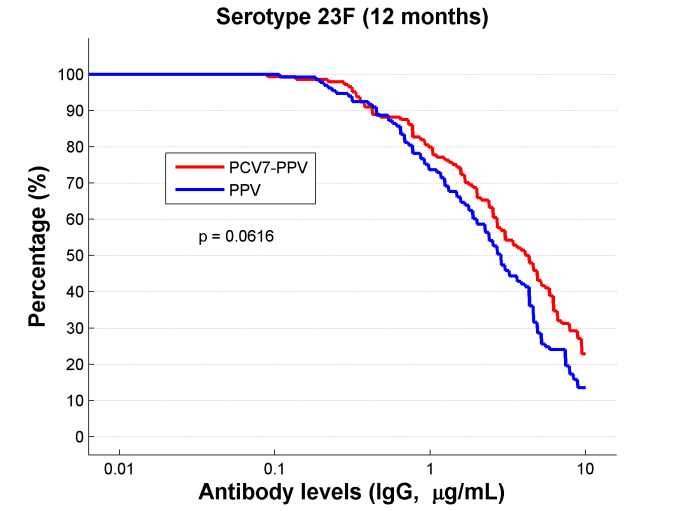 |
