# Supplementary material for: A Randomized Clinical Trial of the Immunogenicity of 7-Valent Pneumococcal Conjugate Vaccine Compared to 23-Valent Polysaccharide Vaccine in Frail, Hospitalized Elderly
Source: PLoS One. 2014 Apr 23;9(4):e94578. doi: 10.1371/journal.pone.0094578 (PMC3997415; doi:10.1371/journal.pone.0094578)
Supplement: Protocol S1 — (PDF) [file pone.0094578.s002.pdf]

TITLE OF PROJECT: The immunogenicity of 7-valent pneumococcal conjugate vaccine (PCV-7) in vulnerable elderly populations at high risk for invasive pneumococcal disease

INVESTIGATORS

Professor C Raina MacIntyre,
Post graduate fellow,
MBBS (Hons 1), FRACP, FAFPHM, M App Epid, PhD
National Centre for Immunisation Research and Surveillance of Vaccine Preventable Diseases,
Children's Hospital at Westmead
Tel: 61 2 98451329 Fax: 61 2 98453095
Email: RainaM@chw.edu.au
Research experience: 15 years of research experience in infectious diseases epidemiology and clinical trials, over 80 peer reviewed publications resulting from that research and over $8 million in competitive research grants.

Professor Richard Lyndley,
Moran Foundation for Older Australians Professor of Geriatric Medicine
MBBS, MD, MRCP, FRCP, FRACP
University of Sydney,
Western Clinical School,
Westmead Hospital
Hawkesbury Road
Westmead NSW 2145
Tel: 9845 5082 Fax: 9687 1429
Email: richardl@westgate.wh.usyd.edu.au
Research experience: Professor Lindley's broad research interests have concentrated on collaborative research in clinical medicine (acute geriatric medicine, stroke and randomised controlled trials). He has been an investigator in many of the trials that have changed clinical practice for older people, such as aspirin for acute ischaemic stroke, blood pressure lowering for secondary prevention, statins for the high risk elderly and currently thrombolysis for stroke. He initiated the start-up phase of the Third International Stroke Trial (2000-2003), evaluating thrombolysis for acute ischaemic stroke, and as Principal Investigator has raised £800,000 (AUD$2,000,000) for the project. He remains Co-Principal Investigator for the current expansion phase of the trial (2003-5). He has been (or is currently) an investigator in a further 16 randomised controlled trials ranging from breast cancer treatment to the prevention of falls.

Associate Professor John Sullivan,
Principal Scientist and Manager of R&D in the Tissue Typing & Research (TT&R) department,
Australian Red Cross Blood Service, Sydney.
BSc (hons), Ph.D., M.P.H..
Australian Red Cross Blood Service
153 Clarence St, Sydney, NSW 2000
Phone: 61-2-92294467 Fax : 61-2-92294521
e-mail: jsullivan@arcbs.redcross.org.au
Research experience: Over 20 years of research experience in the areas of immunogenetics, transplant immunology and the immunology of infectious diseases and over 70 papers in peer reviewed journals.

Professor Peter B McIntyre,

Director, National Centre for Immunisation Research and Surveillance of Vaccine Preventable Diseases
Children's Hospital at Westmead
MBBS (Hons), FRACP, FAFPHM, MPH, PhD
National Centre for Immunisation Research and Surveillance of Vaccine Preventable Diseases, Children's Hospital at Westmead,
Tel: 61 2 98451257 Fax: 61 2 98453095
Email: PeterM@chw.edu.au
Research experience: Over 20 years of research experience. His major areas of research experience have been in invasive Haemophilus influenzae type b/(Hib) disease, pneumococcal disease and pertussis.

Professor Gwendolyn L Gilbert.
Professor/ Director, Centre for Infectious Diseases and Microbiology, Institute of Clinical Pathology and Medical Research, Westmead Hospital, Westmead
MBBS, MD, FRACP, FRCPA
Centre for Infectious Diseases and Microbiology, Institute of Clinical Pathology and Medical Research, Westmead Hospital, Westmead, New South Wales, 2145 Australia;
Tel: 02 9845623, Fax: 02 9893 8659
Email: lyng@icpmr.wsahs.nsw.gov.au,
Research experience: 25 years experience in applied research into diagnosis, epidemiology and prevention of infectious diseases (especially in women, infants and children and vaccine preventable diseases) public health microbiology and microbial epidemiology.

SCIENTIFIC PROTOCOL

Aims: To compare impact of the 7-valent pneumococcal conjugate vaccine (PCV7) with that of the 23-valent polysaccharide vaccine (PPV) in hospitalised adults aged 60 years and over, without a past history of vaccination with polysaccharide vaccine on immunogencity, reactogenicity and nasopharyngeal colonisation with Streptococcus pneumoniae.
Null hypothesis: That PCV7, given alone or followed by a dose of PPV, does not confer any additional benefit to that offered by PPV alone for the same serotypes of S. pneumoniae in hospitalised elderly adults.

Simple Description:
The bacteria pneumococcus (also known as streptococcus pneumoniae) is the most common cause of pneumonia in the community, and a major cause of illness and death in the elderly. Australia has an ageing population. The health and wellbeing of the elderly has been identified as a national priority. The National Health and Medical Research Council (NHMRC) of Australia recommends that adults aged 65 years and over should be immunised with polysaccharide pneumococcal vaccine (PPV). PPV has been available for many years in Australia, but is least effective in those at greatest risk, the sick and the elderly.
A new pneumococcal conjugate vaccine (PCV-7) has been available since the end of 2000, but is currently indicated only for children, because it has never been tested in adults. This vaccine uses different technology, and is conjugated to a protein to make it more effective. Clinicals trials of PCV7 have largely been limited to children aged 0-4 years, and have shown it protects 93.9% of children under 2 years of age against invasive pneumococcal disease (IPD).
Our study aims to look at the efficacy of this new vaccine, currently only registered for children, in the sub-group of the population who are at highest risk for pneumococcal disease - hospitalised elderly. We will vaccinate hospitalised elderly people with PCV or PPV and compare their immune response to the two different vaccines. If PCV is more effective than PPV, this has implications for the development and use of conjuagated pneumococcal vaccines for adults.

Background/Literature Review
Streptococcus pneumoniae is the most common cause of community acquired pneumonia, and a major cause of morbidity and mortality in the elderly. [1]It also causes meningitis, bacteraemia, otitis media, and other infections. The mortality rate for invasive pneumococcal disease in the over 60 age group ranges from 5-57%, with higher mortality in immunosuppressed people, people with underlying chronic diseases and those of advanced age. [2-5] It is a complex organism, as there are over 80 known serotypes. In addition, rates of penicillin and multidrug resistant pneumococcal disease are increasing in Australia and other industrialised countries, [6] so that preventive strategies such as vaccination are increasingly important. The Institute of Medicine released a report titled "Vaccines for the 21st Century" in 2000, where hypothetical vaccines were ranked in order of desirability based on burden of disease, associated costs and likely positive impact on public health and society in general. The most desirable vaccines for human health included those against pneumococcal disease (along with vaccines against cytomegalovirus, influenza, diabetes, multiple sclerosis, rheumatoid arthritis and group B streptococcus). [7]

The next twenty years will see an increase in the Australian population of over 65 year-olds from 12.5% to 18%, and a doubling of those over 85 years old. Pneumonia in the frail elderly will place increasing demands on our health care system, and better prevention will not only relieve much suffering, but has the potential to result in significant cost savings. The National Health and Medical Research Council (NHMRC) recommends that all adults aged 65 years and over should be immunised with 23-valent polysaccharide pneumococcal vaccine (PPV) at least once. [8]Younger persons who have medical risk factors are also recommended for immunisation. PPV has been available in Australia for decades, and has been shown to be cost-effective in these groups. [9] This

vaccine confers protection against 23 pneumococcal serotypes, which cover the majority of invasive disease in adults. However, a meta-analysis of clinical trials has shown that it is most effective in younger, immunocompetent persons. [10]The antibody response to PPV in the elderly and in people with pre-existing medical conditions is less predictable, and observational studies have suggested reduced or no efficacy in the very elderly or immunocompromised. [11, 12] The dilemma associated with the use of PPV is that it is least effective in those at greatest risk of pneumococcal disease and its complications, the sick elderly. [10]

Recently, conjugate technology, whereby a protein is linked to a polysaccharide to enhance immunogenicity and confer immuonologic memory, has been applied to pneumococcal vaccines. The primary aim is to enable young children to mount a protective immune response to pneumococcal polysaccharides, but the improved immunogenicity of these vaccines has the potential to induce improved responses in older persons with impaired immunity and to confer protection of longer duration through memory responses. The currently licensed 7 valent pneumococcal conjugate vaccine (PCV-7) targets the serotypes most common in young children, namely 4, 6B, 7V, 9V, 14, 18C, 19V and 23 F. These serotypes are all also included in the 23-valent PPV, along with 16 other serotypes. Isolates causing invasive disease in adults are less likely to be among the serotypes included in PCV-7. During 2001-2002, in Australia, 1,900 invasive isolates from non-Indigenous adults were submitted for routine serotyping; 69.2% and 76.6% belonged to serotypes and serogroups, respectively, included in the 7-valent vaccine and 96.1% and 96.6% belonged to the 23 serotypes and groups, respectively included in the polysaccharide vaccine. The most common serotypes isolated from adults with IPD (in order of frequency) were 14,4,9V,23F,6B,3,19F,18C,22F,6A,19A,7F,9N,8,11A,1,12F. [13] Thus the top 10 isolates were on cross-reacted with 7 valent serotypes except 3 and 22F. Given the likelihood of suboptimal efficacy data for PPV in sick, elderly populations, it is possible that the lesser serotype coverage of PCV-7 may be more than compensated for by increased immunogenicity and induction of immune memory. Regardless of the ultimate clinical value of PCV-7, if improved immunogenicity compared to PPV can be demonstrated, this has implications for further development and use of conjugated pneumococcal vaccines of higher valency.

PCV7 has been licensed in Australia since the end of 2000. Clinicals trials of PCV7 are limited to children aged 0-4 years, have shown 94% efficacy against invasive pneumococcal disease (IPD) of vaccine serotypes in children under 2 years of age. [14]The serotypes most prevalent in antibiotic resistant pneumococci are also those most common in young children, so it can be expected to have a disproportionate impact on reduction of antibiotic resistant pneumococci. [15] Strain replacement is also an issue of interest, as is the effect of vaccination on nasopharyngeal carriage and herd immunity.

Currently, there are very limited[16] published data internationally on conjugate pneumococcal vaccines in adult populations. In adult renal transplant patients it is more immunogenic than PPV. [17]With its increased immunogencity, it may be a candidate for prevention of pneumococcal disease in vulnerable adults.Two immunogenicity studies are underway in the UK and the US looking at scheduling options and immune responses in healthy adults aged >65 years (personal correspondence, Dr Mary Ramsay Health Protection Agency UK and Dr Cynthia Whitney Centres of Disease Control Atlanta). These groups agree that there is still a need to study the use of conjugate vaccine in sick, elderly populations where high disease burden and sub-optimal PPV responses may justify the use of a substantially more costly vaccine.
The unique contribution of our study will determine the immunogenicity of PCV7 in a vulnerable elderly population with known high incidence of IPD. It is impractical to measure efficacy in such a group in a clinical trial so proxy measures of efficacy using immunologic surrogates is necessary. With increasing demands being placed on our health care system by an ageing population, more effective prevention of IPD in this group could result in significant cost savings. [18]

Methods:
Design: A randomised, clinical trial of PCV7 + PPV compared to 23 valent polysaccharide vaccine (PPV) alone in hospitalised elderly patients.

Subjects/eligibility: Any patient 60 and over years of age admitted under the geriatric, cardiology or rheumatology unit at Westmead hospital, who has not received pneumococcal vaccine, will be eligible. To be admitted under this unit patients must have complex, multi-system pathology, as described below. Patients with a history of pneumococcal vaccination (as determined by self-report and validation by general practitioner records) will be ineligible.

Setting: The Geritaric, Cardiology and Rheumatology units of Westmead Hospital, Sydney. The Westmead Department of Geriatric Medicine has seven consultants and offers an acute emergency geriatric medicine service 24 hours a day. There are between 2,000 and 2,500 admissions a year of 1,500 individual patients (1,300 inpatients and 200 day-patients, some of which have multiple admissions). Westmead Hospital does not have a general internal medicine department and patients are generally admitted to geriatric medicine if they present with a decompensating syndrome (delirium, falls, incontinence or immobility), if they usually live in institutional care or if they are 60 and over years of age and are not considered appropriate for any other speciality, because of multiple pathology, especially cognitive impairment and dementia. Pneumonia is the commonest single reason for admission to geriatric medicine (7%), with respiratory infections, septicaemia and exacerbations of chronic obstructive airway diseases accounting for 300 cases per year. The commonest diagnostic categories are respiratory infection, heart failure, collapse and syncope, stroke, urinary sepsis and septicaemia [data from the Decision Support Unit, Western Sydney Area health Service for financial year 2002/3]. There are 200 deaths a year. All consultants have agreed to their patients being approached for the study. The rheumatology unit admits patients with complex rheumatological and autoimmune disorders, most of whom are on immunosuppressive treatments. They are therefore in the category indicated for pneumococcal vaccine by NHMRC. All consultants in the unit have agreed to their patients being approached for the study. The Cardiology unit is the busiest unit in the hospital. Most patients are admitted with ischaemic heart disease or cardiac failure. This is also an indication for pneumococcal vaccination. All cardiologists have agreed to their patients being approached for the study.

Sample size: A sample size of 132 subjects per vaccine group will enable estimation of reactogenicity and response rates between groups with 95% confidence intervals, as detailed in Table C. The study size will be sufficient to identify a poorly immunogenic or an excessively reactogenic vaccine. For each individual the change in log-22FA levels for each serotype pre-post vaccination will be calculated. The average change will then be compared between schedules and between age groups using t-tests. For the purposes of the sample size calculations the estimated standard deviation for the change in log10-22FA levels within any group is taken as 0.55, which was the average standard deviation observed across serotypes in a UK pilot study (M Ramsay, personal communication).

Approximately 150 subjects are required in each arm, allowing for failure to complete the protocol in 15-20% of each study arm in this vulnerable population. To allow for unexpected additional follow up problems, we will aim to recruit 200 in each arm as follows:

1. PCV7, followed 6 months later by PPV, no past vaccination with PPV	200
2. PPV, no past vaccination with PPV	200

The case fatality rate for admitted inpatients is 15-18%, reflecting the severity of illness and comorbidity in this group. We expect an 80% participation rate, consistent with recruitment to other

trials by this Unit. We estimated that in NSW, where pneumococcal vaccine is recommended but not funded, about 30% of the population aged >65 years will be already vaccinated, based on Victorian data comparing vaccination rates pre-and post funding of the >65 years program.. [19] The study was conceived and funded before the new free pneumococcal vaccine program for the elderly came into being. Early recruitment after the funded pneumococcal vaccination program for adults aged >65 years, which commenced in January 2005, shows that >80% of admitted geriatric patients are vaccinated, making them ineligible for the study. This is the justification for extending the age range to 60-64 years, which then includes patients who are not eligible for free vaccine, and are likely to have baseline vaccination rates of <30%.
Consent: Written informed consent will be obtained from patients who are able to give consent. We will also include people with dementia and cognitive impairment, as it is important not to exclude this group of frail elderly patients. However, such patients will need to have a carer able to make the relevant reactogenicity measures (see below). Guardianship Board approval will be required if patients do not have capacity to consent. This is defined as:

- being capable of understanding the general nature and effect of the trial
- being able to retain the information
- being able to communicate this information to the researchers.

Even when patients are deemed to not have capacity, they can still often contribute to the consent process. Appropriately short and simple trial materials will help in this regard, and the applicants (RIL) have experience in this area. 20 The Westmead Department of Geriatric medicine has extensive experience with consent issues in geriatric clinical trials. [20, 21]The Department is currently recruiting patients in four randomised controlled trials of stroke treatment and has a 10 year history of successful trial recruitment. The recent appointment of a Professor and Senior Lecturer to create a new academic department of geriatric medicine has provided the necessary experience and resources to expand clinical research.

Recruitment and enrolment: The trial nurse will identify all new admissions on a daily basis, and will validate vaccination status from hospital and general practitioner records. This is necessary because a previous Australian hospital-based study in which CIA was involved established that reported vaccination status of hospitalised elderly patients was often unreliable, requiring validation by general practice records. [19]If consent is given by the patient and/or guardian, vaccination will be performed at the end of the hospital stay, to ensure the optimum balance between possible effects on immunogenicity of acute illness and the high costs of out-of –hospital vaccination. This is also relevant as it is likely that such a policy would be most suitable for implementation.

Randomisation: Patients will be recruited, consented, baseline data collected and then randomised by a personal computer which records, in a secure fashion, all patients randomised. Equal numbers of men and women will be recruited, bearing in mind that the unit admits more women than men. We will balance randomisation by age (continuous variable) and gender. Randomisation (on-site by computer) will be secure as baseline details will be recorded prior to treatment allocation of a numbered vaccine vial. The vaccine vials will be blinded to the patient (by a sticker covering the label). Patients will be initially blinded for the 1st vaccine dose, but it will not be possible to blind patients in the long term, since one arm receives a single vaccine and the other arm receives two vaccinations, 6 months apart. The study nurse and CI's will be blinded to the first dose and for assessment of reactogenicity measurement), but the differing presentation of PPV and PCV7 might lead to trial staff being able to differentiate between vaccines, even if the labels are removed. Allocation will be need to be known at 5 months to organise the follow up doss of PPV for subjects who received PCV-7. Laboratory testing will be blinded.

Vaccination procedure: The study nurse will perform immunisation, sample collection and subject follow-up. The vaccination will be deferred if the subject's temperature is above 38C or they have an acute illness. Day of vaccination will be Day 1. Vaccines will be administered by intramuscular

injection in the arm. The nurse will write the subject's initials and date of administration on an adhesive label bearing the subject's study number and stick this onto the empty vaccine container. Pneumococcal vaccine will be given in the non-dominant arm. No other vaccines will be administered concomitantly. Subjects will be observed for 15 minutes after vaccination for any immediate adverse reaction. Standard immunisation practices will be followed and appropriate precautions for any anaphylactic reactions will be taken.

Assessment and notification of reactogenicity: The vaccine study nurse will give the subject or carer a health diary labelled with their study number together with a digital thermometer, pen and ruler. The nurse will provide instructions on how to use the thermometer and how to complete the health diary. Study nurses will complete an adverse event questionnaire by telephone on day 1 and day 7. Any medical consultations, medications taken and hospitalisations will be ascertained at 3 months, 6 months (follow up visit), 9 months and 12 months (follow up visit). The causal relationship between serious events and vaccination will be categorised by the investigators, as one of the following: related; probable; possible; remote or unrelated. Events will be graded as mild, moderate or severe. If appropriate, such events will be notified to the Therapeutic Goods Administation via the "Blue Card"

Collection and testing of samples: Blood samples: At least 10mls but no more than 20mls of blood will be taken. No more than two attempts at venepuncture will be made without the expressed verbal consent of the subject to continue. Failure to obtain a blood sample will not vaccination but will preclude participation in the study. Serum samples will be taken by venepuncture before, and then at 6 and 12 months after vaccination to determine vaccine specific antibody responses. (see below)
Nasopharyngeal samples: A nasopharyngeal swab will be taken at study entry and at the 12 month follow up visit. The trial nurse will be trained in proper nasopharyngeal collection methods.

Main outcomes:
1. Immunogenicity: (see below for details)
2. Reactogenicity: (see below for details)
3. Nasopharyngeal colonisation with S.pneumoniae

Secondary outcome measures – using a subset of 30% of study participants
1. pneumococcal serotype specific IgG avidity after each dose to assess immunological memory
2. functional antibody assays using opsonophagocytosis (to be done at 12 months only)
3. antibody response to the carrier protein pre and post vaccination

1. Immunogenicity:
a) a) geometric mean fold rises (with 95% confidence intervals) of the vaccine serotype specific IgG antibody in all participants.
b) the proportion of subjects achieving a 1.5 fold rise in antibody titre for each pneumococcal serotype

For the PCV7 arm, immunogenicity to PCV7 alone will be measured at 6 months, after which a dose of PPV will be given. At 12 months, the immunogenicity of this PCV7-PPV schedule will be measured. For the PPV arm, immuogenicity to a single dose of PPV will be measured at 6 and 12 months.
We will measure IgG antibody to each of the PCV7 serotypes by ELISA, which has been long established in CIB's laboratory, with appropriate World Health Organization reference standards. We will also measure seroresponse to the 23-valent PPV vaccine serotypes which are most prevalent but not contained in PCV7 (types 3 and 22).

As it is possible that the potency of such antibody if present may be reduced, functional assays- also established with well-validated methodology in CIBs labortatory - will also be performed. The only two international studies currently underway (comparing PCV7 and PPV in healthy adults) are both immunogenicity studies, without clinical endpoints.
This is a longstanding problem, which is recognised in the pneumococcal vaccination literature, due to the rare nature of clinical events. [22] It should be noted that vaccine trials in general are different from therapeutic trials, because the outcomes are rare and the effect of the vaccine is prevention rather than treatment. In fact, increasingly, vaccines are registered on the basis of immunogenicity data alone, in the absence of clinical data. An example is meningocococcal C conjugate vaccine, which has been implemented as a national program in the UK, Australia and the Netherlands on the basis of immunogenicity data alone.

2. Reactogenicity:
We will measure reactogenicity to the two vaccine schedules by telephone follow up, and if required by a visit from the trial nurse. This is defined as
a) the proportions of subjects with significant (>2.5cm) local reactions to study vaccines for seven days following vaccination.
b) the proportions of subjects with pyrexias (>38oC) within 3 days and 7 days of immunisation.

3. Nasopharyngeal colonisation:
A nasopharyngeal swab will be collected from all participants at baseline and 12 months post-vaccination, to ascertain nasopharyngeal colonisation with pneumococcus and the serotypes involved.

Follow up: The study nurse will telephone all patients or their carers at 7 days after vaccination to ask about reactogenicity to the vaccine. Patients will then be followed up at 6 months and 12 months after baseline sera are collected. At these milestones, the study nurse will visit patients in their place of residence to conduct follow up.

Baseline (date of vaccination):	clinical data collection in hospital<br>Serum collection (for serology)<br>Nasopharyngeal specimen<br>Vaccination (PCV& or PPV)
7 days:	Telephone follow up
6 months:	Home visit<br>Clinical data collection<br>Serum collection<br>Vaccination with 23vPPv for PCV arm only.
12 months:	Home visit<br>Clinical data collection<br>Serum collection<br>Nasopharyngeal specimen

Laboratory methods:
Serology: Serum will be collected from patients at baseline, 6 months and 12 months after vaccination. As part of a CRC-Vaccine Technology funded project Dr Sullivan's group has recently completed a study of pneumococcal-specific antibodies in a blood donor population. This involved the screening of over 800 hundred blood donors for any pneumococcal antibodies by ELISA and a further characterisation of the serotype-specific antibodies in a sub-group (~6% of the study population) with an anti-pneumococcal titre greater than a standard pneumococcal reference serum (prepared from a pool of individuals vaccinated against pneumococcus). In addition, functional antibodies (defined by a flow cytometric opsonophagocytosis assay) were also studied against three common disease-causing serotypes (6B, 9V and 23F). This study is in the final stages

of data analysis and it is anticipated that a paper will be submitted shortly. Thus, both gold standard assays for the characterisation of pneumococcal antibodies (ie ELISA and the flow opsonophagocytois assays) are well established in our laboratory.

Pneumococcal antibody ELISA. **A**ntibody levels to each of the seven individual (4,6B,9V,14,18C and 23F) polysaccharide serotypes that are common to the PPV and PCV vaccines will be measured using an enzyme-linked immunosorbent assay (ELISA) according to the consensus protocols. [23, 24] All serum samples (both pre and post vaccination) will be preabsorbed with purified pneumococcal cell wall polysacharride (C-PS) (Serum Statens Institute) and a capsular preparation from 22F [23, 24] and antibody concentrations will be determined for each sample using three serial dilutions (this will be done for each of the seven serotypes) and read off a standard curve prepared using C-PS absorbed 89-SF reference serum. Assay conditions will be optimised [23, 24] for the following: (a) concentration of individual polysaccharides used to coat the ELISA plates, (b) concentration and absorption times for C-PS and 22F and (c) additional individual ELISA reagents as required.
In brief, each antigen is diluted in 0.05 M sodium bicarbonate buffer (pH 9.6), then added to the wells of microtiter plates (Maxisorp; Nunc, Roskild, Denmark) and incubated at 37°C for 5 hrs in a humidified environment (or at 4°C overnight). After each step, the plates are washed with 300 μl/well of phosphate-buffered saline (PBS; pH 7.2) containing 0.1% Tween 20 using an automated plate washer (Wellcozyme 812 SW2) programmed for a 30-s soak and five wash cycles. The assays are performed at room temperature. PBS-Tween 20 containing 2% bovine serum albumin (BSA; Sigma Chemical Co., St. Louis, Mo) is used for blocking non-specific binding (200 μl/well, 2 h incubation) and for further dilutions. All serum samples are tested in duplicate and incubated for 2 h.. Serial dilutions of the C-PS and 22F absorbed patient serum samples and the relevant QC and standard reference standards (note reference serum 89-SF is only absorbed against C-PSare added and the plates incubated for a further 2 hrs at room temperature. The plates are washed 5 times and a dilution of a goat anti-human IgG-alkaline phosphatase conjugate added and the plates again incubated for another 2 hrs at room temperature. After a further 5 washes a diluted substrate (p-nitrophenyl phosphate) is added and the plates incubated at room temperature for an additional 2 hrs (or working substrate solution,20 min incubation). The reaction is then stopped by the addition of 50 ul of 3M NaOH and optical density measured at 405 nm using a 690 nm reference filter [23, 24]. Optical densities will then be converted to antibody concentrations using the CDC ELISA program (available via the CDC, [25]).
Flow cytometric opsonophagocytic assay. The opsonophagocytic assay will be performed using consensus protocols [26, 27] with minor adjustments. Briefly, the seven serotypes of *S. pneumoniae* 4,6B, 9V, 14, 18C and 23F (obtained from ATCC) are plated on 5% sheep blood agar (Oxoid, Australia) and grown over night in a 5% CO_2 atmosphere at 37°C. Isolated colonies displaying α haemolysis are then inoculated into 5 ml Todd-Hewitt broth with 0.5% yeast extract (Beckton Dickinson) and incubated without shaking for 3 h. Bacterial cultures are grown three times to obtain highly encapsulated pneumococci. The bacteria are then pelleted by centrifugation (800 × *g* for 10 min at room temperature) and resuspended in 5 ml of bicarbonate buffer (0.1 M $NaHCO_3$ [pH 8.0]). FAM-SE labelling is performed by adding 50 μl of 5,6-carboxyfluorescein, succinimidyl ester (FAM-SE; Molecular Probes) solution (10 mg/ml in dimethyl sulfoxide [Sigma]) and incubating the mixture for 1 h without shaking at 37°C in 5% CO_2. Following addition of 1 ml of 2% paraformaldehyde (Electron Microscopy Sciences, Fort Washington, PA), cells are fixed over night at 37°C without shaking. Non-viability of the labelled bacteria is confirmed by culturing 100 μl of bacterial suspension on a blood agar plate as described before. FAM-SE labelled bacteria are washed by centrifugation six times in 20 ml of opsonophagocytosis buffer (Hanks balanced salt solution with Ca^{2+} and Mg^{2+} [Life Technologies], 0.2% bovine serum albumin [Sigma] and 1× penicillin-streptomycin [Trace Scientific]), then diluted 1:100 in opsonophagocytosis buffer, and counted using Flow Count (Beckton Dickinson). Bacterial suspension are adjusted to a concentration of 2.0×10^7/ml and stored at – 20°C until used.

HL-60 (human promyelocytic leukemia cell line; CCL240; ATCC) are cultured at a concentration of 1.0×10^5 cells/ml in 90% IMDM containing 1% L-glutamine (Trace Scientific) and 10% foetal calf serum (CSL Bioscience) twice weekly. Differentiation of these cells into granulocytes is achieved using the same medium supplemented with 100 mM N, N-dimethylformamide (Sigma) for a period of up to 7 days with a cell density of $4 \times 10^5 - 6 \times 10^5$. Differentiated HL-60 cells are harvested by centrifugation ($160 \times g$ for 10 min) and then washed twice in 15 ml of opsonophagocytosis buffer. Cells are counted and the concentration adjusted to 2.5×10^6 cells/ml. Prior to flow cytometric opsonophagocytic analysis, serum samples are heated to 56°C for 30 min to inactivate any endogenous complement activity. The flow cytometric opsonophagocytic assay is done in two stages. Initially, the samples are screened for the baseline antibody activity at 1:8 (lowest dilution) in duplicate. In brief, 20 µl of neat serum is transferred to polypropylene titre tubes (Falcon) coated in Sigmacoat (Sigma), then 40 µl of bacterial suspension (2.0×10^7/ml) added and incubated in a shaking water bath at 37°C for 30 min. Then, 20µl baby rabbit complement (Csix, USA) is added to each tube except the HL-60 cell control (to which opsonophagocytosis buffer is added) and incubated at 37°C for 15 min. 80µl (2.0×10^5 cells) of washed, differentiated HL-60 cells are then added and incubated for a further 30 min at 37°C. At the end of this incubation, 160 µl ice cold 0.9% NaCl/0.02% EDTA solution is added to each tube to stop phagocytosis. Samples are then kept on ice prior to analysis. Titre tubes are vortexed for 3 s and 100µl of 0.5% trypan blue (Trace Scientific) added to quench fluorescence of any adherent bacteria. Then titre tubes are placed inside polystyrene tubes for flow cytometric analysis. Three controls are included in each assay: (i) HL-60 control containing only cells and bacteria, (ii) complement control containing all reagents except antibody source, and (iii) a positive control sample (serum from an adult vaccinated with the 23-valent pneumococcal vaccine).
Samples will be analysed using a Beckman Coulter EPICS Elite EPS flow cytometer. A minimum of 5000 gated HL-60 granulocytes was analysed per tube. FAM-SE is excited at wavelength of 488 nm, and the FAM-SE signals of the gated viable HL-60 cells measured at 530 nm. The upper limit of background fluorescence is measured using HL-60 cell controls and consisted of autofluorescence of HL-60 cells. An analysis region is placed above the cell control fluorescence peak and included 98% of the population. A second analysis region is used to determine the percentage of HL-60 cells with a fluorescence greater than the control cells for each dilution. Serum samples with a maximum phagocytic uptake of ≥ 30% will be analysed further. The opsonophagocytic titre of these samples will be evaluated in the second stage. Seven two fold dilutions of test sera, beginning with neat, are made in the round-bottom microtiter plates coated in Sigmacoat (Sigma), then transferred to polypropylene titre tubes, and the flow cytometric opsonophagocytic assay done as described above. The opsonophagocytic titre is defined as the reciprocal of the highest dilution that gives ≥ 50% of the maximum phagocytic uptake. Samples with a maximum phagocytic uptake of < 30% are considered negative and will be reported to have a titre of 4. The presence of significant functional antibody is defined as a titre ≥ 32.
Competitive inhibition will be carried out using homologous and heterologous polysaccharides. Serum samples and a positive control sample (1:16 lowest dilution tested) will be tested for opsonophagocytic antibodies to the 7 pneumococcal serotypes (4,6B, 9V, 14 18C and 23F) after preabsorption for 30 min at room temperature with equal volume of homologous or heterologous polysaccharide diluted to a final concentration of 0.5 mg/ml.

<u>Testing of pneumococcal carriage:</u> Nasophayrngeal swabs will be collected using a calcium alginate on wire swab. Swabs will be inoculated directly on to 5% horse blood agar containing gentamicin 5mg/L, by rolling it over ¼ of the plate's surface. In the laboratory, the inoculum will be streaked on to all 4 quadrants using a sterile loop. Plates will be incubated for 18-24 hours (37oC, in 5% CO2) and then examined for the presence of alpha-haemolytic colonies resembling pneumococci. Species will be confirmed by optochin sensitivity and bile solubility. Growth will be graded as scanty (<25 colonies on quadrant 1 only); 1+ (>25 colonies in quadrant 1 and <25 colonies in

quadrant 2); 2+ (>25 colonies in quadrant 2 and <25 colonies in quadrant 3); 3+ (>25 colonies in quadrant 3 and <25 colonies in quadrant 4) 4+ (>25 colonies in quadrant 4) (O'Brien, 2003). Several colonies resembling pneumococci from each plate will be subcultured and serotyped, using a recently described molecular method (Kong, 2003).

Statistical analysis.
Intention to treat analysis will be done. We will use appropriate corrections to reduce the probability required to accept statistical significance. Each immunological endpoint (eg serology, functional antibodies) will be analysed separately, and correlations between the different endpoints will also measured. Within the sub-group that are to be studied, we will look for correlations between serology and functional antibody for a number of specific serotypes.
Statistical analyses will be carried out using InStat software, version 3 (GraphPad Software, Inc., San Diego, CA) and standardised against the CDC ELISA program. The results will be expressed as geometric mean concentrations (GMC) of IgG antibody to each polysaccharide. Antibody titres will be log transformed prior to analysis and comparison of titres between groups will be made using analysis of variance and regression analysis if required. Fishers exact test and Chi-squared test will be used where appropriate to compare proportions of seroconverters.
Correlation between opsonophagocytic titre and antibody concentration will be calculated using linear correlation coefficient.
Comparisons of local and systemic reactions to routine and study vaccines will be made using Chi-squared and Fishers exact test where appropriate. 95% confidence intervals will also be calculated.
References

ETHICAL ANALYSIS
Potential risks: The major ethical implication of this study is that pneumococcal polysacharide vaccine (PPV) is recommended for all adults aged 65 years and over by the NHMRC. It is estimated that in NSW, where this vaccine is recommended but not funded, that only 30% of the target group are vaccinated. Study subjects will be unvaccinated. All subjects in the PCV7 arm (ie receiving the new vaccine), will also receive PPV one month later. Therefore, no study subject will be denied the recommended vaccine, and all subjects stand to benefit, as they are unvaccinated at the time of enrolment, and would not have received vaccination otherwise. Neither vaccine has any serious side effects. The vaccine being trialled, PCV7, may cause minor local reactions, but there is no evidence that the incidence of these will be any greater than those experienced after the recommended vaccine, PPV.

Potential benefits: The major benefit is that the study will result in unvaccinated elderly people receiving pneumococcal vaccine, which is recommended for this age group. The study may show that in this highly vulnerable population, PCV7 offers better protection against pneumococcal disease than PPV. Pneumococcal diseases is one of the highest ranked priorities in vaccine-preventable causes of human morbidity and mortality globally. Sick, elderly people are at greatest risk of pneumococcal disease and its complications. The next twenty years will see an increase in the proportion of over 65 year olds from 12.5% to 18% of the Australian population, and a doubling of those over 85 years old. Healthy ageing is a national research priority, and vaccination is an important preventive health measure in this age group.
The existing polysacharide pneumococcal disease is least effective in this population. A protein conjugate pneumoccocal vaccine is more immunogenic in children under 2 years (who do not respond to PPV), but only covers 7 serotypes. Protein conjugate pneumococcal vaccines are likely to be more immunogenic in adults and should confer immunologic memory, relevant to the need for repeated doses in adults. No studies have examined nasopharyngeal carriage of pneumococcus following PCV in adults. Our study will allow comparison of response to PCV7 alone to PPV at 6 months, as well as to priming with PCV7 followed by PPV at 12 months. This trial has the potential to increase the local awareness of pneumococcal vaccination. Participation in the trial is likely to

benefit the individual who will receive a recommended treatment which they had not previously been offered.

RESEARCH PLAN

Proposed date of commencement: May 2005
Estimated duration: three years

Care of Participants

This study will not affect the patients's relationship with the hospital. Patient consent forms have been drafted and are attached to this application. Those who agree to participate in the study will be informed of their results and what those results means in relation to their immunity pneumococcal disease. Participants will be encouraged to contact the study investigators if they have any concerns.

Review of Progress:

The study investigators will meet fortnightly to review the study progress. Monthly data reviews and basic analyses will be performed. If one vaccine appears to be statistically significantly favourable over the other, based on immunogenicity data, the study will be prematurely terminated.

Management of Adverse Events

We will measure reactogenicity to the two vaccine schedules by telephone follow up, and if required by a visit from the trial nurse. This is defined as

a) the proportions of subjects with significant (>2.5cm) local reactions to study vaccines for seven days following vaccination.

b) the proportions of subjects with pyrexias (>38oC) within 3 days and 7 days of immunisation.

Local reactions and pyrexias will be treated symptomatically with paracetamol where required. Subjects will also be encouraged to call NCIRS if they think that they have been adversely affected by participation in this trial. If the patient becomes ill or is injured as a result of participation, then reasonable costs of medical treatment will be paid by Westmead hospital and the Children's Hospital at Westmead.

Winding up procedures

The study will terminate 12 months after the last patient is recruited. Patients will be followed up at 7 days, 6 months and 12 months after baseline sera are collected. At these milestones, the study nurse will visit patients in their place of residence to conduct follow up. This study does not involve any longer term, on-going care of participants beyond the 12 months. During the 12 months of follow up, any required care directly as a consequence of the study will be provided by the study investigators at the Westmead Hospital geriatric unit. Feedback from this study to participating patients will be in terms of their immunity to pneumococcal disease.

Access to data

Data will be kept on password protected computer files for the duration of the research. Hard copies of the data will be kept in a locked filing cabinet. Only the study investigators will have access to the computer and paper files. The computer files will be kept long term in a password protected database. All identifying data will be coded and de-identified for participant's privacy.

Will data be collected from a Federal Government agency?

No data will be obtained from any government agency for use in this study.

Storage or disposal of data

Data will be kept on password protected computer files for the duration of the research. The computer files will be kept long term in a password protected database. Hard copies of the questionnaire will be kept in a locked filing cabinet. The paper files will be retained for five years and will be disposed of using a document shredder.

References

1. McIntyre P, Gidding HF, Gilmour R, et al. Vaccine Preventable Diseases and Vaccination Coverage in Australia, 1999-2000. Comm Dis Intell 2002; Suppl May 2002.
2. Watanakunakorn C, Greifenstein A, Stroh K, et al. Pneumococcal bacteremia in three community teaching hospitals from 1980 to 1989. Chest 1993; 103:1152-6.
3. Marrie TJ. Bacteraemic pneumococcal pneumonia: a continuously evolving disease. J Infect 1992; 24:247-55.
4. Pesola GR, Charles A. Pneumococcal bacteremia with pneumonia. Mortality in acquired immunodeficiency syndrome. Chest 1992; 101:150-5.
5. Benenson AS. Control of communicable diseases in man. In: Benenson AS, ed. Washington, DC: American Public Health Association, 1995:495.
6. Collignon PJ, Bell JM. Drug-resistant Streptococcus pneumoniae: the beginning of the end for many antibiotics? Australian Group on Antimicrobial Resistance (AGAR). Med J Aust 1996; 164:64-7.
7. Medicine Io. Vaccines for the 21st Century: tools for decision making. In: Stratton K, Durch JS, Lawrence RS, eds. Washington DC: National Academy Press, 2000.
8. National Health & Medical Research Council. The Australian immunisation handbook. In: Williams A, ed. Canberra: Australian Government Publishing Service, 2003.
9. Sisk JE, Riegelman RK. Cost effectiveness of vaccination against pneumococcal pneumonia: an update. Ann Intern Med 1986; 104:79-86.
10. Fine MJ, Smith MA, Carson CA, et al. Efficacy of pneumococcal vaccination in adults. A meta-analysis of randomized controlled trials. Arch Intern Med 1994; 154:2666-77.
11. Shapiro ED, Berg AT, Austrian R, et al. The protective efficacy of polyvalent pneumococcal polysaccharide vaccine [see comments]. N Engl J Med 1991; 325:1453-60.
12. Butler JC, Breiman RF, Campbell JF, Lipman HB, Broome CV, Facklam RR. Pneumococcal polysaccharide vaccine efficacy. An evaluation of current recommendations. JAMA 1993; 270:1826-31.
13. Watson M, Bayley K, Bell JM, et al. Laboratory surveillance of invasive pneumococcal disease in Australia in 2001 to 2002—implications for vaccine serotype coverage. Comm Dis Intell 2003; 27:478–487.
14. Black S, Shinefield H, Fireman B, et al. Efficacy, safety and immunogenicity of heptavalent pneumococcal conjugate vaccine in children. Northern California Kaiser Permanente Vaccine Study Center Group [see comments]. Pediatric Infectious Disease Journal 2000; 19:187-95.
15. Joloba ML, Windau A, Bajaksouzian S, Appelbaum PC, Hausdorff WP, Jacobs MR. Pneumococcal conjugate vaccine serotypes of Streptococcus pneumoniae isolates and the antimicrobial susceptibility of such isolates in children with otitis media. Clinical Infectious Diseases 2001; 33:1489-94.
16. Molrine DC, Antin JH, Guinan EC, et al. Donor immunization with pneumococcal conjugate vaccine and early protective antibody responses following allogeneic hematopoietic cell transplantation. Blood. 2003; 101:831-6.
17. Kumar D, Rotstein C, Miyata G, Arlen D, Humar A. Randomized, double-blind, controlled trial of pneumococcal vaccination in renal transplant recipients. Journal of Infectious Diseases. 2003; 187:1639-45.
18. Ament A, Fedson DS, Christie P. Pneumococcal vaccination and pneumonia: even a low level of clinical effectiveness is highly cost-effective. Clinical Infectious Diseases 2001; 33:2078-9.
19. MacIntyre CR, Kainer MA, Brown GV. A randomised, clinical trial comparing the effectiveness of hospital and community-based reminder systems for increasing uptake of influenza and pneumococcal vaccine in hospitalised patients aged 65 years and over. Gerontology. 2003; 49:33-40.

20. Donnan GA, Davis SM, Chambers BR, et al. Streptokinase for acute ischemic stroke with relationship to time of administration: Australian Streptokinase (ASK) Trial Study Group. JAMA. 1996; 276:961-6.
21. Anonymous. The International Stroke Trial (IST): a randomised trial of aspirin, subcutaneous heparin, both, or neither among 19435 patients with acute ischaemic stroke. International Stroke Trial Collaborative Group.[see comment]. Lancet. 1997; 349:1569-81.
22. Clemens JD, Shapiro ED. Resolving the pneumococcal vaccine controversy: are there alternatives to randomized clinical trials? Rev Infect Dis 1984; 6:589-600.
23. Wernette CM, Frasch CE, Madore D, et al. Enzyme-linked immunosorbent assay for quantitation of human antibodies to pneumococcal polysaccharides. Clinical & Diagnostic Laboratory Immunology 2003; 10:514-9.
24. Anonymous. Bacterial Respiratory Pathogen Reference Laboratory, 2003.
25. Anonymous. CDC ELISA Program. Vol. 2004: Centers for Disease Control, 2003.
26. Martinez JE, Romero-Steiner S, Pilishvili T, et al. A flow cytometric opsonophagocytic assay for measurement of functional antibodies elicited after vaccination with the 23-valent pneumococcal polysaccharide vaccine. Clinical & Diagnostic Laboratory Immunology. 1999; 6:581-6.
27. Romero-Steiner S, Frasch C, Concepcion N, et al. Multilaboratory evaluation of a viability assay for measurement of opsonophagocytic antibodies specific to the capsular polysaccharides of Streptococcus pneumoniae. Clinical & Diagnostic Laboratory Immunology 1019; 10:1019-24.
